# Consensus among healthcare stakeholders on a collaborative medication therapy management model for chronic diseases in Malaysia; A Delphi study

**DOI:** 10.1371/journal.pone.0216563

**Published:** 2019-05-10

**Authors:** Naeem Mubarak, Ernieda Hatah, Mohd Aznan Md Aris, Asrul Akmal Shafie, Che Suraya Zin

**Affiliations:** 1 Kulliyyah of Pharmacy, Department of Pharmacy Practice, International Islamic University, Kuantan, Malaysia; 2 Lahore Pharmacy College, University of Health Sciences, Lahore, Pakistan; 3 Faculty of Pharmacy, University Kebangsaan Malaysia, Jalan Raja Muda Abdul Aziz, Kuala Lumpur, Malaysia; 4 Kulliyah of Medicine, International Islamic University, Kuantan, Malaysia; 5 School of Pharmaceutical Sciences, University of Sains, Penang, Malaysia; Universite de Bretagne Occidentale, FRANCE

## Abstract

**Background:**

The general problem is lack of inter-professional collaboration and the way private primary care responds to manage chronic diseases in Malaysia. Absence of prescription review, inadequate patient education, the highest percentage of prescribing errors and half of the chronic disease patients are nonadherent. Medicines are the most common and life long used interventions in chronic diseases. Hence, the need to manage medicine in chronic diseases becomes obligatory. As both general practitioner and community pharmacist can dispense medications, this has resulted in a business rivalry. There is a need to build consensus among various healthcare stakeholders for a collaborative medication therapy management model (CMTM) where community pharmacist has an active role in chronic care.

**Method:**

This study utilized modified e-Delphi method to build consensus. A validated e-Delphi survey was administered to a purposive sample of 29 experts. Consensus was pre-defined to be the point where >85% of the experts fall in either agree or strongly agree category for each statement. The inter-expert agreement was computed in both rounds using Intra-class correlation coefficient and Kendall's W. Delphi operates in an iterative fashion till there comes stability in responses. At the end of each round, experts were provided aggregate response, their own response and choice to change their response in the light of aggregate response.

**Results:**

Response rate was 70.73% and 100% in 1^st^ and 2^nd^ round, respectively. Consensus was achieved on 119/132 statements which mainly referred to the need, structural and regulatory aspects of CMTM model in Malaysia. However, there were some flashpoints on dispensing separation and means to finance this model. Stability in response of experts was achieved after 2^nd^ round; hence, no next round was executed.

**Conclusion:**

Overall, the study findings witnessed the expert panel’s support for the CMTM model. Study helped to sketch CMTM model and facilitated development of some recommendations to the authorities which may help to formulate a policy to bring CPs under a working relationship with GPs. Hence, this study should be taken as a call for redefining of the roles of CPs and GPs in Malaysia.

## Background

Chronic diseases pose a huge burden on the Malaysian healthcare system. In Malaysia, primary care developments are slow to offer an efficient chronic care delivery for growingly ageing population [1–5]. World Health Organization (WHO) country profile reveals an alarming burden of chronic diseases in Malaysia where 73% of all deaths were related to chronic diseases [6,7]. Malaysia is among the top ten countries in the world in terms of prevalence of diabetes, for instance, National Health and Morbidity Survey-2015 revealed that Malaysia has highest number of diabetic patients (3.5 million) in the region [3]. ″National survey on the use of medicines by Malaysian consumers-2015″ revealed 70.8% respondents were not satisfied by the current level of counselling in the clinics and expressed the dire need of additional education and counselling to manage their medicine and disease state [8]. Furthermore, recent research has highlighted rampant prevalence of inappropriate prescribing in nursing homes and private clinics in Malaysia [9,10]. A systematic review of 17 studies has placed Malaysia on top in South East Asia with the highest percentage (34%) of prescribing errors [11]. These medications errors increase incidence of adverse drug related admissions in hospitals and contribute a significant burden on healthcare system in Malaysia [12].

The general problem is the way private primary care network functions to manage chronic diseases. Numerous studies have highlighted number of apprehensions on the GPs’ practice as a single care provider where GPs diagnose, dispense, educate, counsel all alone or through a medical assistant or a nurse, which is even worst as they have never been trained in medicines as compared to a pharmacist [13,14]. As mentioned, GP can also dispense medications (which itself constitutes a conflict of interest and reason of overprescribing), there is hardly any prescription reviewed by the community pharmacists (CPs) who are at a conveniently accessible position for public. Malpractices have also been documented in the literature on the side of CPs, such as handling complex medical conditions at pharmacy without referral to a GP, dispense medications which require a prescription, and perform dose adjustments or change of the drug therapy without communicating to a GP.

Thus, there are four main problems in Malaysia, which demand and advocate a shift in the healthcare policy to make a system to optimize and rationalize the medicine use in chronic care, these are:

- over and inappropriate prescribing in private GPs clinic [15] and absence of prescription review in private clinics [16–18].
- lack of patient education and knowledge about how to use or store a medication appropriately, or how to avoid or recognise an adverse effect of a medication or what could be possible interactions with other medications or supplements or traditional medicine a patient is taking, [8,19].
- almost 50% chronic disease nonadherent patients [14] due to absence of any adherence support service in private sector [8,20].
- high prevalence of polypharmacy in older adults with chronic diseases [10].

Medicines are the most common and life long used interventions in chronic diseases. Hence, the need to manage medicine in chronic diseases becomes obligatory. However, it requires an effective medicine management model based on a defined system which dually monitors rational prescribing, quality use of medicines and ensures optimal patient education and adherence support in primary care. If there is a system of medicine management, CPs may utilize their medicine expertise to rationalize the use of medicines among chronic disease patients. Prescription review and adherence support offer a platform which can engage CP and GP in collaborative practice to manage medications. The collaboration between GP and CP have successfully resolved many potential drug related problems and have a proven trajectory of improvements in health outcomes in developed countries [21–25]. However, simply replicating a model which was successful in some developed country might not result the same in Malaysia, as each country has its own ground realities. Thus, studies should first seek opinions and aims to build consensus of all the relevant stakeholders to explore all dimensions and then move ahead. This requires, as a first step, open communication between stakeholders to bring them on same page to optimize patient care.

In this situation, a consensus building approach involving relevant stakeholders or experts would be of significant importance as it ensures that everyone’s interests have been considered along with all the distinct perspectives, concerns and inputs [26,27]. Delphi method represents one of the consensus building methods which has been regarded as a structured communication of experts who can offer valuable contribution in order to resolve a complex issue, and thus used as an efficient tool to help decision making, conflict management and identifying priorities, barriers, concepts and best practices [28].

## Research objectives

The overall objective of this study was to seek consensus among different healthcare stakeholders for a ″collaborative medication therapy management″ (CMTM) model for chronic diseases in Malaysia which may involve CPs and GPs in an active collaboration for management of medicines and diseases.

The specific objectives of this Delphi study were to:

- To identify the perspectives of an expert panel, comprised of healthcare stockholders/experts, on current situation and need of collaboration between CP and GP for a ‘collaborative medication therapy management’ (CMTM) model for chronic diseases in Malaysia.
- To seek and gauge level of consensus on various aspects of CMTM model (e.g., the regulatory requirements, perceived barriers, administrative aspects and means of financing).
- To measure level of consensus among experts on proposed solutions to various flashpoints/problems between different stakeholders on various aspects of CMTM model.
- To proffer consensus based recommendations to relevant stakeholders in Malaysia on way forward to CMTM.
- To draw a working sketch of the CMTM model based on the consensus of healthcare experts in Malaysia.

## Methods

This study was granted ethical approval by the Medical Research Ethical Committee, Ministry of Health, Malaysia (ref: KKM/NIHSEC/P16-1632, NMRR-16-1775-32273) and was also approved by the Research Ethics Committee, International Islamic University Malaysia (ref: IIUM/308/C/1/G1527859).

### Survey time frame

The time frame for two rounds of Delphi surveys spanned from August, 2017 to February, 2018 (including 1st round data analysis and; two and three weeks extension in deadline date to respond to survey for 1st and 2nd round, respectively).

### Modified e-Delphi survey

**Fig 1:**
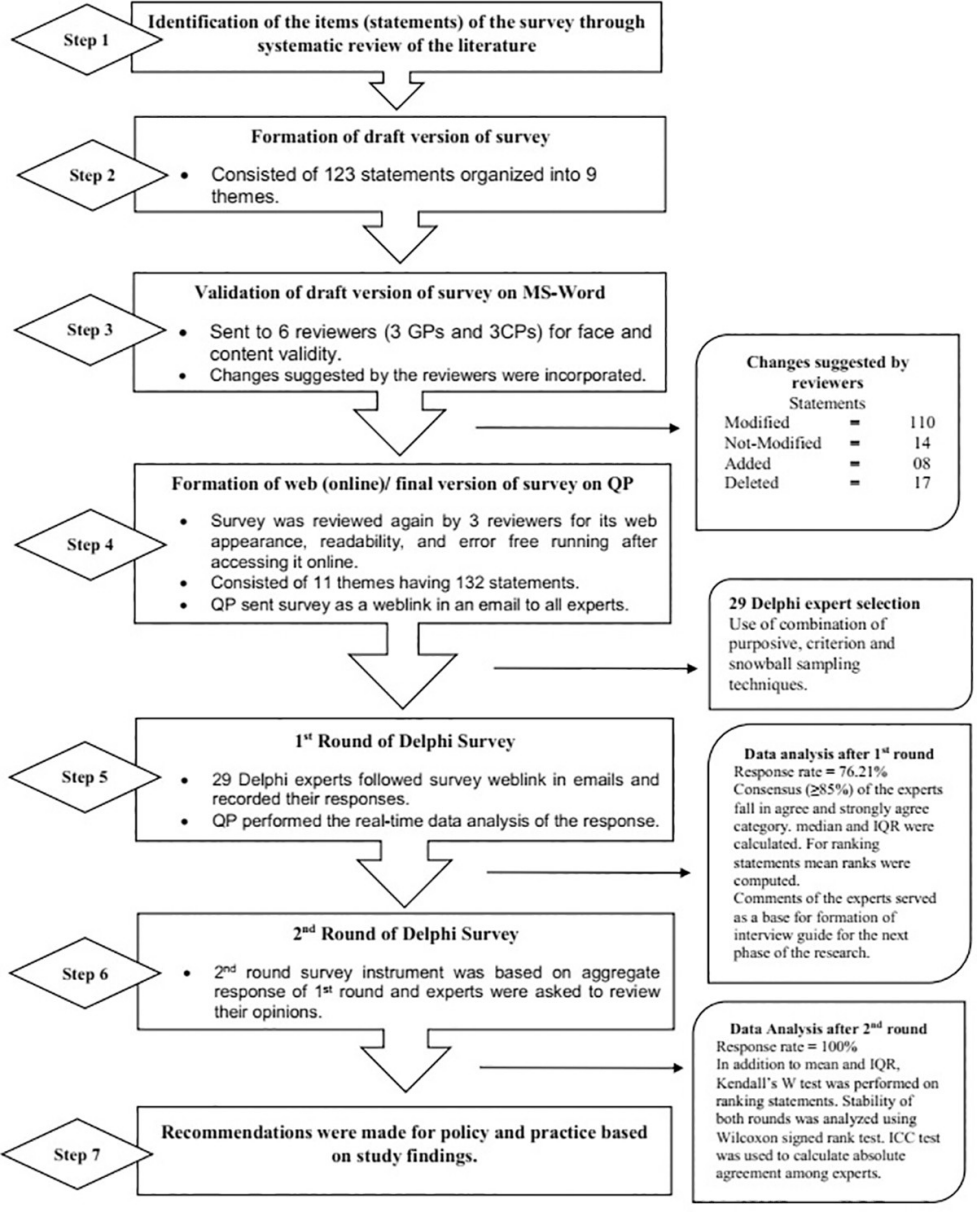
The Delphi process. CP = community pharmacist, GP = general practitioner, CMTM = Collaborative Medication Therapy Management, IQR = interquartile range, ICC = intra-class correlation coefficient, QP = QuestionPro, MS = Microsoft.

This study utilized a modified e-Delphi method, conducted and reported in accordance with the latest guidelines for conducting [29] and reporting [30] a Delphi research. The flow chart of this modified Delphi study is depicted in Fig 1.

### Reasons to choose Delphi method

The research problem under investigation was narrowly defined in Malaysian context. There is a high temperature on the issue in hand, where both stakeholders (GP and CP) hold competing interests (business rivalry as both can dispense because of absence of law for dispensing separation). GPs do not trust clinical skills of CPs while CPs are disappointed being underutilized in patient care. Delphi is appropriate in this situation which involves many stakeholders or there exists a little communication among the stakeholders. Delphi method is free from the bias of dominant individuals who hold superior positions because it does not require experts to meet physically [31] and thus experts remain anonymous and can take part in the survey on their ease (asynchronous participation). Modified Delphi further adds unique advantage to the study for instance, the survey instrument is informed by an extensive review of the literature and thus provide an authentic foundation for 1st round questionnaire [32]. Finally, non-response is generally low in Delphi as pre-research commitments are taken [27,33–35].

### Expert selection

In Delphi context, experts are ″informed individuals, specialists, and those with knowledge about a specific subject ″ [36]. The expert panel was selected across Malaysia. For experts’ selection, we adopted the step by step approach as described in literature [35]. In the first step, this approach involves decision about the categories of experts to be included in the panel based on a pre-set qualifying criteria, followed by identification of names of experts (purposive sampling) and asked initial experts to nominate other experts who might were up to criteria of the study (preparation of knowledge resource nomination worksheet). Finally, nominated experts were ranked based on availability and invited experts through an email. Criteria applied to select the experts for this study (Section A in S2 Appendix) were based on the guidelines [37].

To represent GPs, CPs and Nurses, experts were invited from Ministry of higher education, Ministry of health, various professional associations and councils of these professions, such as Federation of private medical practitioners’ association, Malaysian medical council, Malaysian medical association, Malaysian primary care network, Malaysian pharmaceutical society, Malaysian community pharmacy guild and Malaysian nurses’ association. Thus, a heterogenous expert panel was constituted.

A heterogenous (different experts) panel gives more credibility and acceptance than a homogenous panel, because range of viewpoints may be taken, and thus may cover all the possible aspects of issue in hand. For a heterogenous panel, 5–10 experts per group is the optimal number [38]. As the aim of Delphi survey is not to represent a population, hence, Delphi does not use random sampling to recruit a panel of experts, as unlike conventional survey which generally holds an aim of representativeness [39,40]. To avoid any bias, researcher should set and strictly stick to a pre-defined inclusion criteria. Finally, the results of the study would be considered compromised, if the response rate of the experts falls below 75% during rounds [41].

Experts were included from professional bodies or organizations of GPs and CPs because the proposed CMTM model specifically looking for collaboration between these two stakeholders. We added nurses in this study as they are involved in health care delivery at all point of patient care.

**Fig 2:**
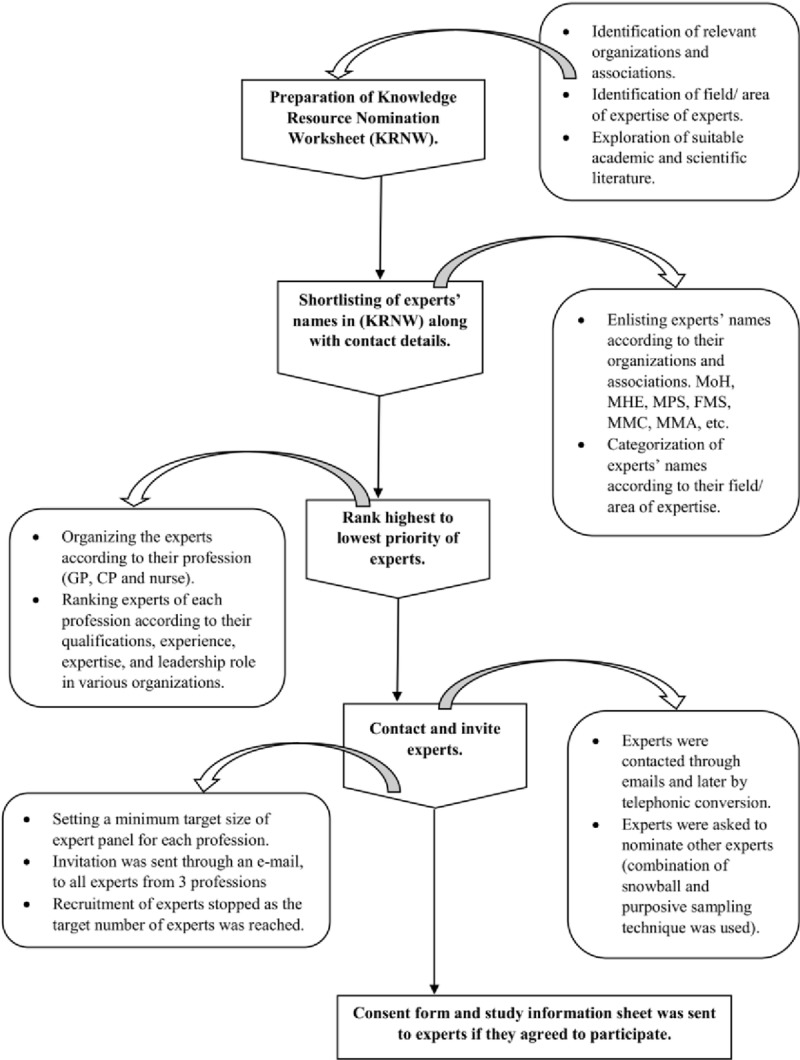
Expert selection flowchart (modified from [35]). MREC = Medical Research & Ethics Committee, KRNW = Knowledge Resource Nomination Worksheet, GP = general practitioner, CP = community pharmacist, MoH = Ministry of Health, MHE = Ministry of Higher Education, MPS = Malaysian Pharmaceutical Society, FMS = Family Medicine Specialist, MMC = Malaysian Medical Council, MMA = Malaysian Medical Association.

Experts were initially approached via email and later by a phone call. They were explained in detail about the study, Delphi method, their responsibilities as a participant in the survey, the time needed to complete the survey and a commitment to complete at least two rounds of the study. They were provided the consent forms and study information sheet which they need to return through email, if they agreed to participate voluntarily. Fig 2 depicts expert selection process.

#### Ensuring optimal response rate

Optimal response rate was ensured by following the recommendations of famous Delphi expert, Okoli [35] which included sending experts, three humble reminders, individualized thank you note, announcing an honorarium of 100 Malaysian Ringgit ($ 24.30), and to hold face to face interviews of the experts between 1st and 2nd round which definitely built rapport and created a personal bond with the experts. We also extended the date of submission by one week and three weeks for 1st and 2nd round, respectively, to maximize the response and to accommodate all the experts.

### Survey instrument

#### Survey development through literature review

The instrument of modified Delphi survey was characterized by directly presenting close ended, pre-determined, well-constructed items (statements) to the expert panel for rating or ranking. The items in modified Delphi survey instrument were informed mainly by the literature review where similar collaboration between CP and GP (in different developed countries) were cited. The detailed search strategy and the articles (which were used to draft the items/statements in Delphi survey) are presented in Section A to C in S1 Appendix. The statements were modified in Malaysian context and assembled in the form of a theme to cover a specific aspect of CMTM model. In draft version, there were nine themes which covered nine specific aspect of CMTM model.

#### Survey instrument validation

The draft version was sent as an email attachment to 6 reviewers (3 CP, 3 GP; not part of panel of experts) to provide feedback for the face and content validity. All the six reviewers were PhD in their respective disciplines and have vast experience of survey instrument making and designs. The reviewers were requested to rate all the statements of the survey using a 4-point Likert scale from ″highly relevant″ to ″non-relevant″. An empty column was also provided in Word document for any comments to improve the statements. Experts were asked to comment, delete or edit any item. The modifications made were incorporated accordingly to bring improvements in the survey instrument. This review of the draft enhanced the clarity in phrasing, eliminated any leading or double barrel questions and improved relevance of the questionnaire.

We used, content validity index/universal agreement method to measure content validity as recommended in the literature [42]. Section D in S1 Appendix-I depicts the detailed version of all the review process and presents all 138 statements into four categories based on reviewers’ recommendations i.e., statements modified, not-modified, added or deleted. Any statement with a CVI less than 0.7 was omitted. Finally, the survey emerged with 132 statements assembled in 11 themes.

#### Web version of the survey (online version)

QuestionPro

After the process of face and content validity on the Word document, the final version of survey instrument was uploaded on the World Wide Web by using an online, flexible and secure survey tool ″QuestionPro″ (QP). QP generates a separate code for each expert to make sure one response per expert. The feature of real time data analysis (analysis as soon as any expert fill the survey) in QP saved a lot of time. QP gave freedom to export the survey results in various downloadable formats for further data analysis for instance, as Statistical Package for Social Scientists (SPSS) file, file for Excel (XML), etc. Furthermore, another unique feature of QP was ″spotlight report″ which assisted experts to view their own response in comparison with collective response of all experts in the panel after the completion of each round. Before sending to the expert panel, weblink of survey was sent to three of the six reviewers to complete the full survey online and provide feedback about the efficiency of the online version, report any missing or non-functional link or unexpected error and to check the type of data received for downloading. There were few problems reported by the experts on the online version and after a formal go ahead the final online version was sent to experts.

Sections of the survey instrument

The final web survey had 4 sections as it appeared online in the sequence given below:

1. A brief introduction of the study; with background and objectives clearly stated.
2. Instructions for experts on ″how to fill the survey″, such as if an expert missed to give response over any statement, the QP was programmed to show a “validity error” on the screen mentioning the missing area highlighted in red to be easily identified by the expert. Similarly, at the end of each theme, QP gave three options to the experts to: ″provide any comment (qualitative feedback) ″, ″Save and continue later″ (to complete the survey in multiple sitting) or ″Next″ to move to the next theme in the survey. In case of confusion or ambiguity expert would just click on the terminology or word given, a pop-up window would appear at the top right side of the webpage and had complete definition and context in which the term was utilized in the survey. These instructions supported the participants to complete the survey with ease and clarity.
3. Demographic details of experts.
4. Survey statements (arranged in 11 themes). The fourth part was the main section of the survey. Out of 11 themes, 6 themes (theme 1, 2, 3, 7, 8 and 10) had statements to be rated on a five points Likert-scale (from strongly disagree to strongly agree) while, 5 themes (theme 4, 5, 6, 9 and 11) had statements to be ranked based on the priority or feasibility of various aspects of CMTM model in Malaysian setting.

Complete survey is attached as Section E in S1 Appendix.

Finally, survey instrument was sent to all experts in the form of an openable weblink, embedded in a separate email generated through QP. The link when clicked by the expert opened on QP interface.

The Delphi process including survey instrument making is given in Fig 1.

#### Instrument administration

After ethical approval, all experts received the survey instrument in the form of an invitation email.

### Defining consensus

It is a fact that a few problems can achieve an absolute 100% agreement among experts. Generally, the consensus is defined by an arbitrary percentage of experts who agree to a statement in a survey, but that percentage must be pre-defined and declared in the survey instrument before the start of the survey [43].

Literature defined consensus as the "gathering of individual evaluations around a median response, with minimal divergence" [44–46]. Another aspect to cross check the point of consensus is the ″stability″ in responses of experts between two consecutive rounds, which must be ensured before concluding iteration in Delphi process [45].

The percentage of agreement is a measure of how many experts agree to a statement and is simply calculated by the number of experts rating ″agree or strongly agree″ to a given statement, divided by the total number of experts. However, for inter-expert agreement, there are various statistical measure, such as median, IQR, Kendall’s W, and ICC. It is recommended to use combination of statistical parameters to report a consensus and not merely relying on the percentage of experts who choose ″agree″ or ″strongly agree″ [47].

The final level of consensus would be measured at the end of 2nd round. The consensus was decided to be accomplished if a statement would receive sum of the percentage of ″agree″ ″strongly agree″ or ″disagree″ ″strongly disagree″ rating by ≥85% of the experts. Thus,

- ≥85% high consensus
- 80% to 84% moderate consensus
- 75% to 79% low consensus
- ≤74% poor or no consensus

For the ranking statements the Kendall’s coefficient of concordance (Kendall’s W), was used to measure the extent of consensus among the experts, in addition to mean rank.

It is important to know when to stop iterations (rounds). Too early stop may contribute to meaningless results and too long would make the process tedious and bring factor of fatigue as bias. Thus, as per literature guidelines, two parameters were planned which would act as indicators to stop the Delphi process in this study [40], they were:

- When the pre-defined consensus level was achieved.
- When there was an evidence of no change of response of experts in two consecutive rounds as defined by Wilcoxon signed rank test of stability.

### 2^nd^ round’s survey instrument

The instrument for 2nd round followed the same administrative procedures. It was identical to 1st round’s instrument in terms of statements, however, with some additional features provided in MS Word document, these were:

- Instructions to complete the survey in the 2nd round.
- Expert’s own response on the given statement in 1st round.
- Collective group response (response of 29 experts) for each items of the survey, in terms of percentage of people who were in favour (agree/strongly agree) or against (disagree/strongly disagree) a statement in the survey.

Thus, the objective of the 2nd round was the re-evaluation of the statements of instrument by the experts in the light of aggregate response, and reasoning of other experts in the group. It would be sent to all the same experts through an email attachment. At the end of 2nd round, the statements where pre-defined level of consensus could not be achieved would be omitted.

### Data analysis

As Delphi operates in an iterative fashion (rounds), data were collected, analysed and shared repeatedly with the experts to review in each round [34]. Literature does not pose hard and fast rules on the exact number of rounds which should be carried out but offers two general stop criteria for iteration as mentioned earlier i.e., stability in response of experts between two consecutive rounds and achieving pre-defined consensus level. If 1st round’s questionnaire is based on literature review, two rounds are sufficient [28,48,49].

Data would be analysed through various statistical operations which are discussed in following sections:

#### Quantitative data

All the statistical operations were performed using the Statistical package for social science (SPSS, version 23.0) by exporting the data files of QP in SPSS.

Rating statements

For rating statements in the survey, Likert scale is generally considered as ordinal, hence, tests were applied accordingly. The results were interpreted through descriptive statistics i.e., median, inter quartile range (IQR). Median measures central tendency of the ratings of the experts while IQR is used to check the dispersion. The reason to choose median and IQR was, both are not affected by extreme values and use of both operations are favoured in literature [50].

Ranking statements

For the ranking themes (where experts were asked to rank or prioritize certain items based on feasibility in Malaysia), the mean and priority rank along with median and IQR were computed for each item. QP would automatically compute the mean rank, also called whole rank based on the ranks given by all 29 experts for each item (statement). This helped in creating the order of the statements based on their priority for each theme. Lower the mean rank, the higher the priority rank. Another test was computed for ranking statements i.e., Kendall’s W (coefficient of concordance) test, which calculated agreement among raters for these statements. Different values of W, for instance, less than 0.3, 0.3 to 0.5, 0.5 to 0.7 and 0.7 to 0.9 would be interpreted as weak, moderate, good and strong agreement, respectively.

Intra-class Correlation Coefficient (ICC)

ICC, two-way mixed model (Type A) test was applied over all the themes (both rating and ranking) individually to check the absolute agreement among the experts. This test is suitable for use when the multiple scores or responses of a respondent must be tested to check agreement among raters. We chose absolute agreement instead of individual agreement during the computation of this test as described in [51]. The interpretation of ICC values are as follows: less than 0.3, 0.3 to 0.5, 0.5 to 0.7 and 0.7 to 0.9 indicate as weak, moderate, good and strong agreement, respectively.

Finally, after the completion of both rounds, to authenticate the decision of consensus, Wilcoxon signed rank test was executed on all items of the survey to compute stability of response of experts between two rounds. The null hypothesis (H0) was ″there is no difference in response of respondents between the two rounds″, while the alternative hypothesis (HA) was ″there is a difference in response of respondents between the two rounds″.

Hence, when p > 0.05, null hypothesis would be accepted and there would be no difference between the Delphi rounds.

#### Qualitative data and narrative synthesis

The comments made by the experts (if any) would be narrated and utilized to inform the interview guide in the next phase of the study. These comments are provided in Section B in S2 Appendix.

### Planning to overcome the limitations of Delphi

Many of the limitations of Delphi, as narrated in literature [52], were managed through a pre-planning which countered various kind of biases in the current study.

Section C in S2 Appendix describes these limitations and how we planned to tackle them.

## Results

### Experts

#### Response and completion rate

Response and completion rate of both rounds of Delphi for all expert categories is detailed in Table 1.

**Table 1:** Response and completion rate of experts in 1^st^ and 2^nd^ round.

Rounds	Category of experts	Invited (n)	Agreed (n)	Completed (n)	Response Rate (%)	Completion Rate (%)
**1**^**st**^ **Round**	GP	16	14	11	68.75	78.57
	CP	11	11	10	90.91	90.91
	Nurse	11	10	8	72.73	80.00
	**Total**	**38**	**34**	**29**	**76.32**	**85.29**
**2**^**nd**^ **Round**	GP	11	11	11	100%	100%
	CP	10	10	10	100%	100%
	Nurse	8	8	8	100%	100%
	**Total**	**29**	**29**	**29**	**100%**	**100%**

% = percentage, n = number of experts, CP = community pharmacist, GP = general practitioner.

#### Demographics of expert panel

**Fig 3:**
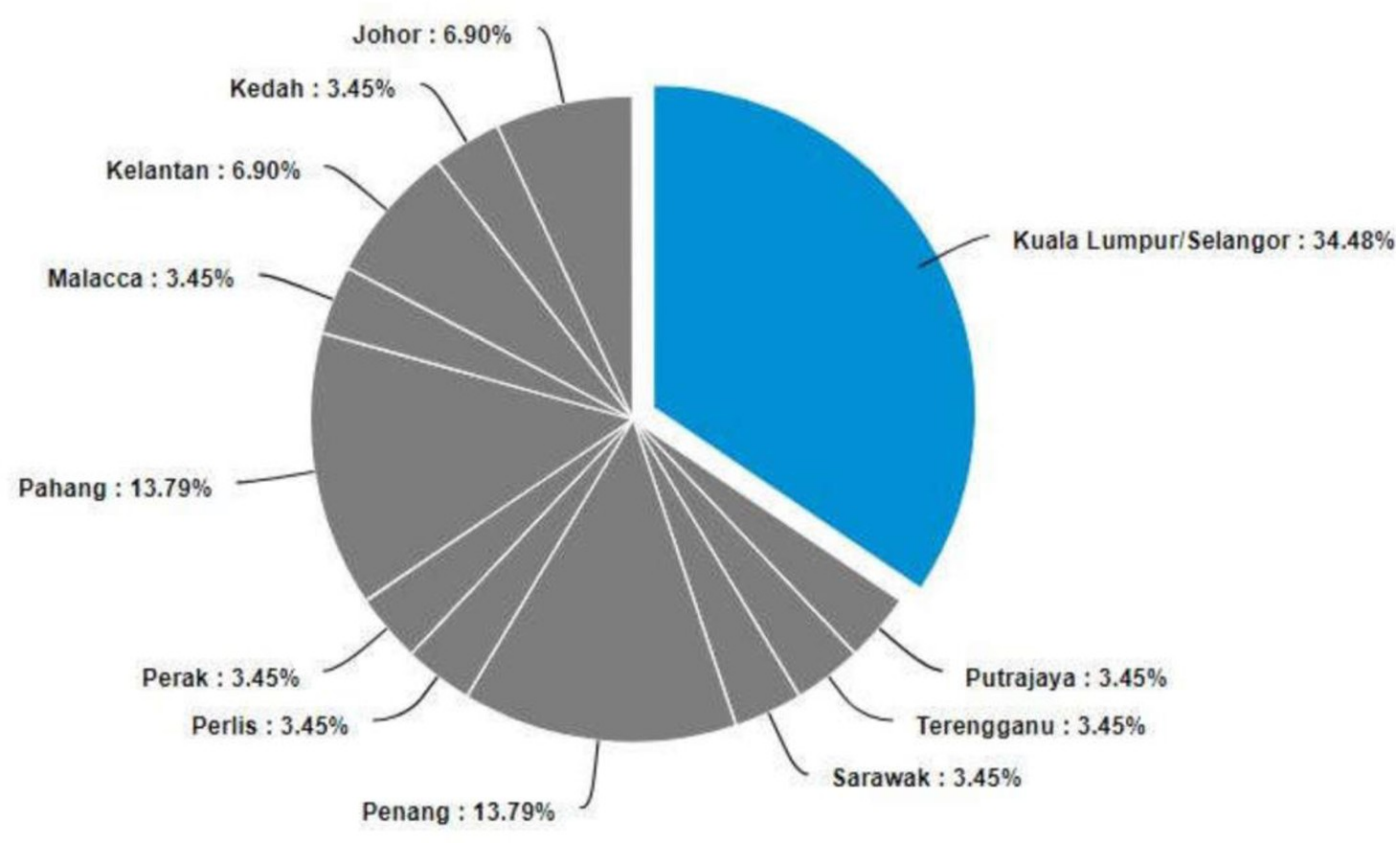
Geographical diversity of Delphi experts in this study across Malaysia.

The expert panel represents a wide coverage across Malaysia, and experts participated from almost all states as depicted in the Fig 3.

There were equal number of male and female experts in the panel. The median number of years of experience of the experts was 24 years with a range (17–39). The demographic data are provided in Table 2, Fig 3 and Section D in S2 Appendix.

**Table 2:** Demographics of Delphi experts.

Characteristics	Category	n (%) where, n_t_ = 29			
		GP (n = 11)	CP (n = 10)	Nurse (n = 8)	Total
**Gender**	Male	5 (17.2)	7 (24.1)	3 (10.3)	15 (51.72)
	Female	6 (20.7)	3 (10.3)	5 (17.2)	14 (48.28)
**Does your training's curricula include inter-professional collaborative practice?**	Yes	7 (24.1)	3 (10.3)	7 (24.1)	17 (58.62)
	No	4 (13.8)	7 (24.1)	1 (3.4)	12 (41.38)
**Where did you get your training (i.e., education and experience) in your related field from?**	Local	5 (17.2)	5 (17.2)	5 (17.2)	15 (51.72)
	Both Local and International	6 (20.7)	5 (17.2)	3 (10.3)	14 (48.28)
**Have you ever worked professionally with a CP?**	Yes	8 (27.59)	-	8 (27.59)	16 (55.17)
	No	3 (10.34)	-	-	3 (10.34)
	-	-	10 (34.48)	-	10 (34.48)
**Have you ever worked professionally with a GP?**	Yes	-	8 (27.59)	8 (27.59)	16 (55.17)
	No	-	2 (6.9)	0	2 (6.9)
	-	11 (37.93)	-	-	11 (37.93)
**If you are in academia; which category do you fall into?**	Professor	2 (6.9)	2 (6.9)	0	4 (13.79)
	Associate Professor	4 (13.79)	1 (3.45)	1 (3.45)	6 (20.68)
	Assistant Professor	2 (6.9)	0	3 (10.34)	5 (17.24)
	Not in academia	3 (10.34)	7 (24.14)	4 (13.79)	14 (48.27)
**Highest Qualification/Degree**	PhD	2 (6.9)	3 (10.34)	3 (10.34)	8 (27.59)
	Specialization (MRCP)	1 (3.45)	-	-	1 (3.45)
	MD	2 (6.9)	-	-	2 (6.9)
	Master	6 (20.69)	2 (6.9)	5 (17.24)	13 (44.83)
	Bachelor	0	5 (17.24)	0	5 (17.24)
Age*	-	52 (45–59)	50 (45–62)	46 (40–55)	50 (40–62)
**Total experience (number of years)** *	-	24 (17–32)	27.5 (20–39)	21 (17–27)	24 (17–39)

*Median (Range) is given for age and total experience.

GP = general practitioner, CP = community pharmacist, n_t_ = total number of experts, n = number of experts.

### Survey instrument

#### Validity of the instrument

Average item level Content Validity Index (CVI) was 0.92, while, Scale level CVI/Universal agreement was computed to be 0.83. Hence, achieved an excellent agreement for both item and scale level validity index.

#### Delphi rounds

The average time taken by the experts to complete the 1^st^ round of survey was 44 minutes and 10 seconds. For themes with rating scale (strongly disagree to strongly agree), in the final round (2^nd^ round) consensus was achieved on:

- 72% (n = 13/18) statements of theme-1,
- 78% (n = 7/9) statements of theme-2,
- 100% (n = 16/16) statements of theme-3,
- 69% (n = 9/13) of theme-7,
- 50% (n = 3/6) statements of theme-8 and
- 82% (n = 28/34) statements of theme-10.

The theme-4,5,6,9 and 11 were not related to Likert scale but were ranking in nature and hence their computation of consensus was carried out through Kendall’s W.

The consensus level among experts for rating statements in 1^st^ and 2^nd^ round based on the percentage of agreement and the stability in responses in two consecutive rounds are given in Table 3. Similarly, consensus level based on median, IQR and Kendall’s W for ranking statements is given in Table 4. Finally, the inter-expert agreement computed through ICC is in the Table 5. The process of reaching consensus for rating and ranking statements are separately depicted in Section E in S2 Appendix, respectively. The statements over which consensus was not achieved after 2^nd^ round are given in Section F in S2 Appendix.

**Table 3:** Consensus among experts in both rounds (based on percentage sum of agree and strongly agree) and stability in response of experts between rounds.

Theme	Sr. No.	Statements	Round	Median (IQR)	(%) (A+SA)	P/ F	Wilcoxon p-value
**THEME 1** **(Need, current practices, and potential disadvantages of lack of CP-GP collaboration)**	1.	Currently in Malaysia, there is no collaboration between CP and GP for patient-centered care services (e.g., CMTM) for chronic disease(s).	1^st^	4 (1)	86.21	P	0.976
			2^nd^	4 (1)	86.21	P	
	2.	The new models of patient-centered community pharmacy services (which involve close collaboration between CP and GP) may help in reducing the incidence of drug related problems in patients with chronic disease(s).	1^st^	5 (1)	79.31	F*	1.00
			2^nd^	5 (1)	86.21	P	
	3.	In Malaysia, the potential of CP in delivering patient-centered care (through CMTM services) is underutilized, leading to resource wasting.	1^st^	4 (2)	75.86	F*	0.648
			2^nd^	4 (1)	82.76	F**	
	4.	Taking example from developed countries, Malaysia should utilize the CP’s potentials in delivering patient-centered care services.	1^st^	5 (1)	89.66	P	0.808
			2^nd^	5 (1)	89.66	P	
	5.	Considering the needs of aging population, it is the right time to focus on patient-centered collaborative care practice between CP and GP in Malaysia.	1^st^	5 (1)	96.55	P	0.739
			2^nd^	5 (1)	96.55	P	
	6.	National survey on the use of medicines by Malaysian consumers, 2012 implied on the dire need for patient education in view of high percentage (56%) of patients who were not aware of the proper use and common side effects of their medications	1^st^	5 (1)	89.65	P	0.796
			2^nd^	5 (1)	89.65	P	
	7.	In the current practice of primary health care model, lack of patient’s motivation is one of the reasons of poor compliance to medicines in chronic disease(s).	1^st^	4 (0)	82.76	F*	1.00
			2^nd^	4 (0)	89.66	P	
	8.	To achieve therapeutic goals, there is a need of motivation to improve patient compliance with the medications used in chronic diseases.	1^st^	5 (1)	96.55	P	0.637
			2^nd^	5 (1)	96.55	P	
	9.	In the current primary health care model in Malaysia, there is no ″prescription review″ process which may serve as a second security layer to alarm prescription errors or inappropriate medicine use.	1^st^	4 (1)	82.76	F*	0.860
			2^nd^	4 (1)	86.21	P	
	10.	When ″prescription review″ will be embedded in system, many drug related problems/errors would be preventable.	1^st^	5 (1)	100	P	0.782
			2^nd^	5 (1)	100	P	
	11.	Absence of ″prescription review″ process may increase the risk for prescriber’s malpractice, such as over prescribing of medications to increase profit margin.	1^st^	5 (1)	89.65	P	0.973
			2^nd^	5 (1)	89.65	P	
	12.	Absence of CP-GP collaboration is disadvantageous for the patients, because patients lose out a protective layer on prescribing (i.e., prescription review by CP).	1^st^	4 (1)	89.66	P	0.973
			2^nd^	4 (1)	89.66	P	
	13.	Absence of CP-GP collaboration is disadvantageous for the individual patient, because of limited education he receives from a single care-provider (GP) due to high number of patients in GPs’ clinics.	1^st^	4 (2)	72.42	F*	0.904
			2^nd^	4 (2)	68.97	F**	
	14.	Without collaborative practice, CPs and GPs may not have the advantage of utilizing each other's expertise in patient-care.	1^st^	4 (1)	93.11	P	1.00
			2^nd^	4 (1)	93.11	P	
	15.	Absence of collaboration between CP and GP is disadvantageous for the Government, because patients miss the proper education on medication use and/or disease management, which may result in wastage of healthcare resources, for example, due to hospitalization and emergency department visits.	1^st^	5 (1)	89.65	P	0.614
			2^nd^	5 (1)	89.65	P	
	16.	In a broader view, GP and CP share common objectives in patient care.	1^st^	5 (1)	96.55	P	1.00
			2^nd^	5 (1)	96.55	P	
	17.	In Malaysia, growing health related problems associated with aging population & chronic diseases can be addressed more effectively by a well-structured patient-centered collaborative practice (in the form of CMTM) between CP and GP.	1^st^	5 (1)	93.1	P	1.00
			2^nd^	5 (1)	93.1	P	
	18.	In Malaysia, CP and GP should work together in managing chronic disease(s).	1^st^	5 (1)	93.11	P	0.963
			2^nd^	5 (1)	93.11	P	
**THEME 2** **(Potential roles and responsibilities of CP)**	19.	CP may enhance GP’s evidence-based medicine practice by providing them important information on medicine use, such as its benefits and risks.	1^st^	4 (1)	89.65	P	0.936
			2^nd^	4 (1)	89.65	P	
	20.	CP may have role in the prescription review which involves identifying and preventing prescription or prescribing errors, such as related to drug interactions or any contraindication.	1^st^	5 (1)	96.55	P	0.808
			2^nd^	5 (1)	96.55	P	
	21.	CP may have role in suggesting GP on alteration in patients’ drug therapy	1^st^	4 (1)	82.76	F*	0.833
			2^nd^	4 (1)	86.21	P	
	22.	CP may have role in advising GP on cost-effective prescribing.	1^st^	5 (1)	89.65	P	0.981
			2^nd^	5 (1)	89.65	P	
	23.	Compared with GP’s assistant, CP can provide patients a more rational advice on the use of medicines based on his training and knowledge of pharmacology.	1^st^	5 (1)	96.55	P	1.00
			2^nd^	5 (1)	96.55	P	
	24.	CP may counsel patients about what to expect from their medicine including its expected pharmacological effects and side- effects.	1^st^	4 (1)	96.56	P	1.00
			2^nd^	4 (1)	96.56	P	
	25.	CP may help in improving patients’ compliance/adherence to medicines by providing an adherence plan to patients.	1^st^	5 (1)	96.55	P	0.803
			2^nd^	5 (1)	96.55	P	
	26.	CP may dispense repeat prescriptions for a patient as per agreed protocols and contacting the GP if a problem arises.	1^st^	5 (1)	96.55	P	0.967
			2^nd^	5 (1)	96.55	P	
	27.	CP may perform dosing adjustments to a patient’s medicine using agreed protocols established with GP.	1^st^	4 (1)	82.76	F*	0.948
			2^nd^	4 (1)	86.21	P	
**THEME 3** **(Potential impact of CMTM)**	28.	CMTM service would improve CP-GP effective communication about patient’s drug therapy	1^st^	4 (1)	96.56	P	1.00
			2^nd^	4 (1)	96.56	P	
	29.	The two-way communication (established under CMTM) between CP-GP would result in improved patient care.	1^st^	5 (1)	82.76	F*	0.805
			2^nd^	5 (1)	86.21	P	
	30.	Collaborative practice like CMTM offers GP to utilize CP’s expertise in pharmacotherapy.	1^st^	5 (1)	93.1	P	1.00
			2^nd^	5 (1)	93.1	P	
	31.	CMTM service offers patients to take benefits from CP’s drug expertise at a highly accessible position (community pharmacy).	1^st^	5 (1)	93.1	P	1.00
			2^nd^	5 (1)	93.1	P	
	32.	CMTM service may improve patient’s clinical outcomes (through its medication therapy review and patient action plan development).	1^st^	4 (1)	89.65	P	0.564
			2^nd^	4 (1)	89.65	P	
	33.	CMTM service may improve patient’s knowledge on self-management of disease (i.e., self-care; how to avoid adverse event or exacerbation).	1^st^	5 (1)	89.65	P	0.627
			2^nd^	5 (1)	89.65	P	
	34.	CMTM service may improve patient’s knowledge on rational use of medicines.	1^st^	4 (1)	89.66	P	0.627
			2^nd^	4 (1)	89.66	P	
	35.	CMTM may significantly reduce hospital/emergency admissions by improving patients’ understanding on disease and its management.	1^st^	4 (1)	79.31	F*	1.00
			2^nd^	4 (1)	86.21	P	
	36.	CMTM practice may encourage cost-effective prescribing which may reduce patients’ cost of treatment.	1^st^	4 (1)	93.11	P	0.851
			2^nd^	4 (1)	93.11	P	
	37.	Patient’s personal medication record (in CMTM) may serve as an early warning system to alarm CP about any under or over-use of medicine by patient.	1^st^	4 (1)	89.66	P	1.00
			2^nd^	4 (1)	89.66	P	
	38.	CMTM service will include a ″prescription review″ process by CP, which may help to ensure patients’ safety by preventing any prescription or prescribing error.	1^st^	5 (1)	100	P	1.00
			2^nd^	5 (1)	100	P	
	39.	CMTM service may reduce medicine waste by improving patient’s compliance to their medicine.	1^st^	4 (1)	89.66	P	0.851
			2^nd^	4 (1)	89.66	P	
	40.	CMTM like service by CP in Malaysia could help patients to better manage their medicine.	1^st^	4 (1)	93.11	P	0.378
			2^nd^	5 (1)	93.1	P	
	41.	In Malaysia, involving CP in CMTM would be an appropriate way to prevent human resource waste i.e., the underutilized CP.	1^st^	4 (1)	82.76	F*	0.599
			2^nd^	4 (1)	86.21	P	
	42.	In Malaysia, involving CP in collaborative practice would help them in their own professional development.	1^st^	4 (1)	93.1	P	1.00
			2^nd^	4 (1)	93.1	P	
	43.	In Malaysia, CP-GP collaboration in CMTM will create opportunities of transition to a value-based health care delivery system.	1^st^	4 (1)	93.1	P	1.00
			2^nd^	4 (1)	93.1	P	
**THEME 7** **(Administrative/surveillance issues of (CP-GP) collaborative practice.)**	44.	Protocol and terms of collaboration between CP and GP must be drafted and agreed beforehand.	1^st^	5 (1)	96.55	P	1.00
			2^nd^	5 (1)	96.55	P	
	45.	CP and GP must be accredited (by their respective regulatory bodies) to provide the collaborative service to patients.	1^st^	5 (1)	93.1	P	1.00
			2^nd^	5 (1)	93.1	P	
	46.	The service would only be provided after approval of Ministry of Health and/or Pharmaceutical division.	1^st^	4 (1)	79.31	F*	0.820
			2^nd^	4 (1)	86.21	P	
	47.	Patients’ recruitment in the CMTM service needs to be done through a referral system (GP to CP and vice versa), for example, through a formal patient’s referral letter.	1^st^	5 (1)	79.31	F*	0.672
			2^nd^	5 (1)	86.2	P	
	48.	Participation in the CMTM service must be with patients’ consent.	1^st^	5 (1)	93.1	P	0.796
			2^nd^	5 (1)	93.1	P	
	49.	Phone may be the best way to communicate for such collaborative practice.	1^st^	4 (1)	62.07	F*	0.934
			2^nd^	4 (1)	62.07	F**	
	50.	In addition to phone communication, at least one monthly face to face CP-GP meeting should be necessary.	1^st^	4 (1)	72.41	F*	0.724
			2^nd^	4 (1)	75.86	F**	
	51.	The communication between CP-GP should always ensure protection over patients’ private and confidential information.	1^st^	5 (1)	96.55	P	0.813
			2^nd^	5 (1)	96.55	P	
	52.	CMTM collaborative services should be allowed only for patients with chronic disease(s), such as hypertension, asthma and diabetes.	1^st^	4 (3)	55.17	F*	0.527
			2^nd^	4 (3)	62.07	F**	
	53.	As a start CPs and GPs should be allowed to recruit only a certain number of patients into the service in a year.	1^st^	4 (2)	75.87	F*	0.658
			2^nd^	4 (1)	79.31	F**	
	54.	CP and GP must allow practice of sharing important patients’ information to each other.	1^st^	4 (1)	79.31	F*	0.847
			2^nd^	4 (1)	89.66	P	
	55.	CP must document all the consultations and/or interventions performed.	1^st^	5 (1)	96.55	P	0.822
			2^nd^	5 (1)	96.55	P	
	56.	CP must communicate all interventions to GP on a structured CP’s interventions form.	1^st^	5 (1)	96.55	P	0.822
			2^nd^	5 (1)	96.55	P	
**Theme 8** **(Chronic diseases where CMTM services may contribute)**	57.	Hypertension	1^st^	5 (1)	96.55	P	1.00
			2^nd^	5 (1)	96.55	P	
	58.	Asthma/COPD	1^st^	5 (1)	96.56	P	1.00
			2^nd^	5 (1)	96.56	P	
	59.	Diabetes	1^st^	5 (1)	96.55	P	1.00
			2^nd^	5 (1)	96.55	P	
	60.	Depression	1^st^	4 (2)	51.72	F*	0.793
			2^nd^	4 (2)	58.62	F	
	61.	AIDS	1^st^	3 (2)	34.48	F*	0.981
			2^nd^	3 (2)	34.48	F	
	62.	Cancer	1^st^	3 (3)	41.38	F*	0.762
			2^nd^	3 (3)	41.38	F**	
**Theme 10** **Proposed solutions for: Problem-1** **Role clarity**	63.	There should be a ''CP-GP Collaborative Practice Agreement'' that defines roles, jurisdictions and terms of CP-GP collaborative practice.	1^st^	5 (1)	96.55	P	0.796
			2^nd^	5 (1)	96.55	P	
	64.	Existence of formal agreements would be an effective way for the development and maintenance of successful collaboration.	1^st^	4 (1)	96.56	P	0.782
			2^nd^	4 (1)	96.56	P	
	65.	These agreements will prevent concerns regarding role encroachment.	1^st^	4 (1)	96.56	P	0.782
			2^nd^	4 (1)	96.56	P	
	66.	These agreements will prevent concerns regarding delivery of contradictory messages to the patients	1^st^	5 (1)	96.55	P	0.822
			2^nd^	5 (1)	96.55	P	
	67.	These agreements should be established, both at professional organizations' level (Malaysian Pharmaceutical Society and Malaysian Medical Association) and at an official level (Ministry of Health).	1^st^	5 (1)	93.1	P	0.952
			2^nd^	5 (1)	93.1	P	
**Theme 10** **Proposed solutions for: Problem-2** **Lack of trust**	68.	Collaboration between CP & GP can be initiated by mutual role recognition and respect.	1^st^	5 (1)	100	P	0.796
			2^nd^	5 (1)	100	P	
	69.	Regular communication between CP and GP may help in building rapport and trust.	1^st^	5 (1)	100	P	0.796
			2^nd^	5 (1)	100	P	
	70.	There should always be direct communication (face to face, telephone, email) between CP and GP about patients (communication should not be passed through patient to avoid misunderstanding).	1^st^	5 (1)	96.55	P	0.822
			2^nd^	5 (1)	96.55	P	
	71.	Joint (CP-GP) continuing professional education’s event or training may strengthen the collaborative service.	1^st^	4 (1)	93.11	P	0.830
			2^nd^	4 (1)	93.11	P	
	72.	Inter-professional module/course should be embedded as a mandatory component in health professional’s degree curriculum.	1^st^	5 (1)	100	P	1.00
			2^nd^	5 (1)	100	P	
**Theme 10** **Proposed solutions for: Problem-3** **Qualification**	73.	Before starting the service, CP should officially get a mandatory accredited training/diploma/course on CMTM service for a specific chronic disease (asthma, diabetes, hypertension).	1^st^	4 (1)	97.11	P	0.842
			2^nd^	4 (1)	93.11	P	
	74.	Accreditation must include evaluation of CP’s competencies, such as clinical knowledge and communication skills (with both GP and patients).	1^st^	4 (1)	96.55	P	0.973
			2^nd^	4 (1)	96.55	P	
	75.	CP’s accreditation training/diploma/course would be a joint venture of Malaysian Pharmaceutical Society and Malaysian Medical Association under regulations of Ministry of Health, Malaysia.	1^st^	4 (1)	93.1	P	0.837
			2^nd^	4 (1)	93.1	P	
	76.	Accredited CP may be called ″consultant pharmacist″ or ″Community Pharmacist Practitioner″, would only be eligible to join these collaborative services for public.	1^st^	4 (1)	89.66	P	0.976
			2^nd^	4 (1)	89.66	P	
	77.	Such accreditation should be renewed after a certain duration that deems appropriate.	1^st^	4 (1)	82.76	F*	0.812
			2^nd^	4 (1)	86.21	P	
	78.	The curriculum for undergraduate pharmacy degree should be made compulsory to include module for patient-centered collaborative practice, i.e., CMTM.	1^st^	5 (1)	100	P	1.00
			2^nd^	5 (1)	100	P	
	79.	After accreditation, continuing professional development/education for CPs may help to further boost their confidence to participate in the changing paradigm in health care delivery (CMTM practice).	1^st^	5 (1)	96.55	P	0.819
			2^nd^	5 (1)	96.55	P	
**Theme 10** **Proposed solutions for: Problem-4** **Compromised Privacy**	80.	Pharmacy should have a counselling room or adequate private consultation space to conduct the CMTM service.	1^st^	4 (1)	89.65	P	0.806
			2^nd^	4 (1)	89.65	P	
	81.	Community pharmacy needs to have appropriate national standards against which CMTM service provision and the clinical care provided could objectively be judged. This would help public to make its own judgement about what to expect of the “best” services, when they visit any pharmacy.	1^st^	4 (1)	96.55	P	0.782
			2^nd^	4 (1)	96.55	P	
	82.	Community pharmacy should be officially categorized and advertised for its scope and area of practice (type of services it offers, i.e., essential and advance).	1^st^	4 (1)	86.21	P	0.858
			2^nd^	4 (1)	86.21	P	
	83.	An electronic national prescription database system should be developed under Ministry of Health to store the prescription records of all the chronic disease patients.	1^st^	5 (1)	93.1	P	0.825
			2^nd^	5 (1)	93.1	P	
	84.	The pharmacy offering CMTM service must have all the means which enables it to connect to national prescription database system to store prescriptions record, such as computer, server, data storage software and internet.	1^st^	5 (1)	93.1	P	0.833
			2^nd^	5 (1)	93.1	P	
**Theme 10** **Proposed solutions for: Problem-5** **Workload**	85.	High dispensary workloads of CP (involved in CMTM service) can be reduced by delegating clerical tasks (e.g., patient identification, appointment scheduling, billing) to pharmacy technicians/clerical staff.	1^st^	4 (1)	96.55	P	0.957
			2^nd^	4 (1)	96.55	P	
	86.	The service should be granted to CP with a pre-set number of patients seen per year based on CP’s ability to cater the service, such as manpower and infrastructure.	1^st^	4 (0)	82.76	F*	0.782
			2^nd^	4 (0)	82.76	F**	
	87.	In Malaysia, pharmacists are adequate to cater the public health needs.	1^st^	3 (2)	48.28	F*	0.545
			2^nd^	4 (2)	58.62	F**	
**Theme 10** **Proposed solutions for: Problem-6** **Consumers perception**	88.	Campaign such as ″Know your medicine by asking your pharmacist″ may help to increase public awareness of the appropriate use of medicines.	1^st^	4 (1)	89.66	P	0.768
			2^nd^	4 (1)	89.66	P	
	89.	To promote public awareness about the importance of CMTM, Government should run a national level campaign to explain the advantages of collaborative practice between CP & GP.	1^st^	5 (1)	96.55	P	0.813
			2^nd^	5 (1)	96.55	P	
	90.	CP should be adequately compensated for providing CMTM services.	1^st^	4 (1)	82.76	F*	1.00
			2^nd^	4 (1)	86.21	P	
	91.	A standard fee structure that is deem appropriate for collaborative CMTM should be determined by authorities, such as Malaysian Pharmaceutical Society and Ministry of Health, Malaysia to avoid profiteering.	1^st^	4 (1)	93.1	P	0.830
			2^nd^	4 (1)	93.1	P	
	92.	The burden of additional consultation fee for CMTM services may be minimized by Government subsidies.	1^st^	4 (2)	75.86	F*	0.653
			2^nd^	4 (1)	79.31	F**	
**Theme 10** **Proposed solutions for: Problem-7** **Policy implementation**	93.	The Government should support a pilot study to evaluate effectiveness, potential issues and concerns raise for community pharmacy based CMTM service.	1^st^	5 (1)	96.55	P	0.655
			2^nd^	5 (1)	96.55	P	
	94.	After the pilot study, to avoid any economic setbacks, the CMTM can be gradually implemented in major cities in Malaysia.	1^st^	4 (1)	82.76	F*	0.467
			2^nd^	4 (1)	89.66	P	
**Theme 10** **Proposed solutions for: Problem-8** **Dispensing separation**	95.	Collaboration between CP and GP can be achieved even without dispensing separation as it does not matter where a patient is getting medicines because at the end he would be seeing a CP.	1^st^	2 (2)	37.93	F*	0.821
			2^nd^	2 (2)	37.93	F**	
	96.	The objective of this collaboration is not to emphasize on selling drugs to make profits but to improve public health outcomes and reduce cost of therapy.	1^st^	5 (1)	96.55	P	0.822
			2^nd^	5 (1)	96.55	P	

CP = community pharmacist, GP = general practitioner, CMTM = Collaborative Medication Therapy Management, COPD = Chronic Obstructive Pulmonary Disease, AIDS = Acquired Immune Deficiency Syndrome, IQR = interquartile range, UHC = Universal Health Coverage, P = pass, F = fail, Q1 and Q3 = quartiles, A = agree, SA = strongly agree, % = percentage. Passed statements are those where conditions of consensus met as described in methods.

*F denotes the statements that failed to reach consensus (≥85%) in 1^st^ round.

**F denotes the statements that failed to reach consensus (≥85%) in 2^nd^ round.

**Table 4:** Consensus among experts in both rounds for ranking statements and stability in response of experts between rounds for mean rank and priority order.

Theme	Sr. No.	Statements	Round	Median (IQR)	Mean Rank	Priority order	Wilcoxon p-value
**Theme 4** **(GP perceived barriers)**	1.	Such collaborations are threat to GP’s job (ruin the clinic business).	1^st^	2 (3)	3.1	1	0.740
			2^nd^	2 (3)	3.03	1	
	2.	CP’s interventions will be projected as a challenge to GP’s clinical decisions.	1^st^	3 (5)	4.0	2	0.858
			2^nd^	3 (5)	4.07	2	
	3.	Such collaborations will lead to violation of GPs jurisdiction.	1^st^	5 (4)	4.76	3	0.636
			2^nd^	5 (4)	4.69	3	
	4.	There will be potential for overlapping roles between GP and CP.	1^st^	5 (5)	5.21	4	0.755
			2^nd^	5 (5)	5.21	4	
	5.	CPs are more product than patient-oriented.	1^st^	6 (4)	5.59	5	0.861
			2^nd^	6 (4)	5.59	5	
	6.	Concerns regarding liability over patient’s information (in shared responsibility).	1^st^	5 (5)	5.62	6	0.858
			2^nd^	5 (5)	5.62	6	
	7.	CPs do not have the appropriate training in providing patient-oriented care services.	1^st^	6 (4)	5.97	7	0.958
			2^nd^	6 (4)	5.97	7	
	8.	GPs do not have time to discuss patient-related medicine issues with CP.	1^st^	7 (6)	6.0	8	0.593
			2^nd^	7 (6)	6.0	8	
	9.	Concern regarding patients’ privacy in community pharmacy setup.	1^st^	8 (5)	7.0	9	0.948
			2^nd^	8 (5)	7.0	9	
	10.	Malaysia does not have enough CPs to cater population health care needs.	1^st^	9 (4)	7.76	10	0.793
			2^nd^	9 (4)	7.83	10	
	**Kendall’s W**		1^st^	**0.2**			
			2^nd^	**0.2**			
**Theme 5** **(CP perceived barriers)**	1.	Collaboration between CP and GP cannot be achieved without ″dispensing separation″.	1^st^	1 (2)	1.83	1	0.248
			2^nd^	1 (1)	1.76	1	
	2.	Lack of trust and appreciation from GP.	1^st^	3 (2)	2.72	2	0.893
			2^nd^	3 (2)	2.72	2	
	3.	GP does not consider CP’s advice as important.	1^st^	3 (3)	3.38	3	0.584
			2^nd^	3 (3)	3.45	3	
	4.	Lack of incentive/remuneration for CPs for providing the service.	1^st^	3 (2)	3.59	4	0.566
			2^nd^	3 (2)	3.59	4	
	5.	CPs have no time because of heavy workloads of other tasks, such as managing the shop and staff.	1^st^	5 (3)	5.55	5	0.893
			2^nd^	5 (3)	5.55	5	
	6.	CPs are not ready to advance their roles in collaborative practice.	1^st^	6 (3)	5.97	6	0.887
			2^nd^	6 (3)	5.97	6	
	7.	CPs do not have expertise to offer such services.	1^st^	7 (2)	6.41	7	0.792
			2^nd^	7 (2)	6.41	7	
	8.	CPs are comfortable with their current roles.	1^st^	7 (3)	6.55	8	0.887
			2^nd^	7 (3)	6.55	8	
	**Kendall’s W**		1^st^	**0.6**			
			2^nd^	**0.6**			
**Theme 6** **(Consumer perceived barriers)**	1.	Consumers are still GP-centered and may not prefer to approach CP.	1^st^	2 (1)	1.93	1	0.617
			2^nd^	2 (1)	1.93	1	
	2.	Consumers are not aware of CP’s such advance roles.	1^st^	3 (2)	2.34	2	0.780
			2^nd^	3 (2)	2.34	2	
	3.	Increased cost to consumers due to additional CP’s consultation.	1^st^	2 (2)	2.38	3	0.839
			2^nd^	2 (2)	2.38	3	
	4.	Consumers will not trust CPs professional training and skills to perform such services.	1^st^	4 (1)	3.34	4	0.744
			2^nd^	4 (1)	3.34	4	
	**Kendall’s W**		1^st^	**0.2**			
			2^nd^	**0.2**			
**Theme 9** **(Chronic diseases in order of priority where (CMTM) service should be started as a first step.)**	1.	Diabetes	1^st^	4 (1)	1.55	1	0.816
			2^nd^	4 (1)	1.55	1	
	2.	Hypertension	1^st^	3 (1)	2.1	2	0.651
			2^nd^	3 (1)	2.1	2	
	3.	Asthma/COPD	1^st^	1 (1)	2.76	3	0.642
			2^nd^	1 (1)	2.76	3	
	4.	Depression	1^st^	5 (1)	4.48	4	0.793
			2^nd^	5 (1)	4.48	4	
	5.	Cancer	1^st^	2 (2)	4.86	5	0.683
			2^nd^	2 (2)	4.86	5	
	6.	AIDS	1^st^	5 (2)	5.24	6	0.670
			2^nd^	5 (2)	5.24	6	
	**Kendall’s W**		1^st^	**0.7**			
			2^nd^	**0.7**			
**Theme 11** (M**eans of remuneration or compensation for CP for CMTM service, that could be more feasible in Malaysian setting.**)	1.	Universal health coverage	1^st^	2 (2)	2.34	1	0.405
			2^nd^	2 (2)	2.24	1	
	2.	Third party payer/health insurance coverage	1^st^	2 (1)	2.45	2	0.834
			2^nd^	2 (1)	2.45	2	
	3.	Direct billing/ Fee for service	1^st^	3 (4)	3.34	3	0.718
			2^nd^	3 (3)	3.45	3	
	4.	Pay for performance	1^st^	4 (2)	4.0	4	0.762
			2^nd^	4 (2)	4.0	4	
	5.	Cost sharing	1^st^	4 (2)	4.1	5	0.691
			2^nd^	4 (2)	4.1	5	
	6.	Capitation	1^st^	6 (1)	6.38	6	0.650
			2^nd^	6 (1)	6.38	6	
	7.	Bundle payment	1^st^	7 (2)	6.62	7	0.965
			2^nd^	7 (2)	6.62	7	
	8.	Incident to service	1^st^	7 (2)	6.76	8	0.867
			2^nd^	7 (2)	6.76	8	
	**Kendall’s W**		1^st^	**0.6**			
			2^nd^	**0.6**			

CP = community pharmacist, GP = general practitioner, CMTM = Collaborative Medication Therapy Management, COPD = Chronic Obstructive Pulmonary Disease, AIDS = Acquired Immune Deficiency Syndrome, IQR = interquartile range, UHC = Universal Health Coverage, Q1 and Q3 = quartiles, Kendall’s W = Kendall’s coefficient of concordance.

**Table 5:** Intra-class correlation coefficient (ICC) test results.

Intra-class correlation coefficient (ICC)										
Themes	Round 1					Round 2				
	Value	95% CI	p-value	F-test	Verdict	Value	95% CI	p-value	F-test	Verdict
**Theme 1**	0.62	0.364–0.821	<0.001	3.35	Good	0.62	0.371–0.823	<0.001	3.34	Good
**Theme 2**	0.54	0.159–0.859	<0.001	2.96	Good	0.50	0.100–0.842	<0.001	2.68	Good
**Theme 3**	0.19	-0.071–0.535	<0.001	1.57	Poor	0.13	-0.127–0.482	<0.001	1.35	Poor
**Theme 4**	0.87	0.712–0.961	<0.001	6.98	Excellent	0.86	0.724–0.963	<0.001	7.28	Excellent
**Theme 5**	0.98	0.942–0.994	<0.001	35.61	Excellent	0.98	0.943–0.994	<0.001	36.39	Excellent
**Theme 6**	0.90	0.642–0.993	<0.001	7.68	Excellent	0.90	0.642–0.993	<0.001	7.68	Excellent
**Theme 7**	0.82	0.662–0.935	<0.001	7.01	Excellent	0.81	0.638–0.930	<0.001	6.40	Excellent
**Theme 8**	0.96	0.886–0.993	<0.001	36.35	Excellent	0.96	0.882–0.992	<0.001	34.37	Excellent
**Theme 9**	0.99	0.965–0.998	<0.001	63.58	Excellent	0.99	0.965–0.998	<0.001	63.58	Excellent
**Theme 10**	0.87	0.798–0.925	<0.001	9.44	Excellent	0.87	0.791–0.922	<0.001	9.17	Excellent
**Theme 11**	0.98	0.943–0.994	<0.001	36.36	Excellent	0.98	0.944–0.994	<0.001	37.17	Excellent

Type A; ICC using an absolute agreement definition using a two-way mixed model, Interpretation of results as: Poor agreement < 0.5, Moderate agreement = between 0.5 and 0.75, Good agreement = between 0.75 and 0.9, Strong agreement> 0.9. CI = confidence interval.

#### Theme-1

Theme-1 of the Delphi survey aimed to map the consensus of experts on the need, current situation, and potential disadvantages of lack of CP, GP collaboration and proposed CMTM model for chronic diseases. It had 18 statements, 13/18 statements, reached consensus in 1^st^round, while, 3 out of the 5 conflicted statements achieved consensus in 2^nd^ round whereas 2 statements failed to reach consensus at this point. The ICC value of 0.62 (<0.001) showed good agreement among experts for both rounds for this theme.

#### Theme-2

Theme-2 evaluated experts’ perspective on the potential roles and responsibilities of CP, if they were to provide a CMTM service in collaboration with GP. This theme involved 9 statements, out of which experts had consensus on 7 in 1^st^ round. The 2 conflicted statements touched the pre-defined consensus level in 2^nd^ round. The ICC result showed good agreement among experts for theme 2 with a value of 0.5 (<0.001) for both rounds.

#### Theme-3

Theme-3 sought expert consensus on potential impact of CMTM service. The theme consisted of 16 statements, out of which 13 statements reached consensus in 1^st^ round, while, 3 statements received consensus in 2nd round. The ICC values pointed to a poor agreement among experts for theme 3 with a value of 0.19 and 0.13 (<0.001) for 1^st^ and 2^nd^ round, respectively.

#### Theme-4

Theme-4 involved 10 ranking statements pertaining to GPs’ perceived barriers on way to collaboration with CPs. The highest rank (most relevant barrier) was obtained by the statement ″CP-GP collaboration is a threat to GP’s job″, which gained a mean rank 3.03 at the end of 2^nd^ round. As the scale used was set to mark highest rank at 1 and lowest rank at 10, any statement, with the lowest mean rank, would indicate the highest priority. The 2^nd^ highest rank barrier was the statement ″CPs’ interventions will be projected as challenge for GP’s clinical decisions″ which receive a mean rank value of 4.07 at the end of 2^nd^ round. Similarly, the third highest ranked barrier was related to concerns regarding the jurisdiction violation, which received a mean rank value of 4.69 at the end of 2^nd^ round. However, the least ranked barrier with a mean rank value of 7.83 after round 2, was ″Malaysia does not have enough CPs to cater population healthcare needs″. The lowest priority or relevance to this barrier indicated that experts are fully aware of the current situation in terms of number of CPs in Malaysia and did not consider it as a barrier anymore. The ICC value of 0.87 and 0.86 (<0.001) denoted excellent agreement among experts for theme 4.

#### Theme-5

Theme-5 also utilized ranking statements to identify the CP’s perceived barriers for a CMTM model in Malaysia. Lack of dispensing separation was the highest ranked barrier, which received a mean rank value of 1.76. The lowest ranked barrier by experts, from CPs’ view point was ″CPs are comfortable with their current roles″ which received the mean rank value of 6.55 after 2^nd^ round and pointed that experts were fully aware of the awakening or realization in pharmacist community in Malaysia about their potential for extended roles. The ICC depicted excellent agreement among experts for this theme with a value of 0.98 (<0.001).

#### Theme-6

Theme-6 highlighted consumer’s perceived barrier if there would be CP’s provided medicine management services in Malaysia. The highest ranked barrier from the consumer’s point of view was ″Consumers are still GP-centred″ with a mean rank of 1.93, while the lowest ranked barrier was ″Consumers will not trust CPs″ which received a mean rank of 3.34. This ranking by experts witnessed that public perception is not a top barrier in Malaysia. Excellent agreement among experts was witnessed by the ICC for this theme with a value of 0.90 (<0.001).

#### Theme-7

This theme was characterized by 13 statements related to administrative or regulatory aspects of CMTM model. By the end of 2^nd^ round 9/13 statements reached consensus level. The ICC test resulted in excellent agreement among experts for this theme with a value of 0.82 (<0.001).

#### Theme-8

In theme-8, various chronic diseases (which could be benefitted by CMTM model) were enlisted. Consensus was achieved on3 diseases which were predicted to be benefitted most significantly by CP-GP collaboration as per Malaysian population health care needs, named as hypertension, asthma/ COPD and diabetes. The ICC value authenticated an excellent agreement among experts for this theme with a value of 0.96 (<0.001).

#### Theme-9

Theme-9 asked experts to rank various chronic disease in order of priority for Malaysian healthcare system. Diabetes, hypertension and asthma were ranked as 1^st^, 2^nd^ and 3^rd^ priority respectively in Malaysia. The ICC value was in range of excellent agreement among experts for this theme with a value of 0.99 (<0.001).

#### Theme-10

Theme-10 sought experts’ opinion on proposed solutions for various barriers and problem CMTM model may encounter during its course. Solutions for problem-1, 2, 3, 4 and 5 received consensus of the experts in the 1^st^ round which did not change by the end of 2^nd^ round.

For problem-6, 4 out of 5 solutions reached consensus at the end of study, however, solution ″burden of additional consultation fee for CMTM services may be minimized by Government subsidies″ could not reach consensus, which hinted that experts in Malaysia did not consider government subsidies as a viable financing options to compensate CP for the CMTM service.

Both solutions offered for problem-7 received experts’ consensus by the end of 2^nd^ round. Problem-8, the solution ″collaboration between CP and GP can be achieved even without dispensing separation as it does not matter where a patient is getting medicines because at the end, he would be seeing a CP″ failed to reach consensus level, as only 37.93% of experts agreed to this. This is very important as it means majority of the experts were of view that collaboration between CP and GP, without dispensing separation would not make sense and would be functionally meaningless. This finding was quite important for this study. The ICC test depicted excellent agreement among experts for this theme with a value of 0.87 (<0.001).

#### Theme-11

Theme-11 enlisted various means of financing the CMTM model and asked experts to rank the most applicable means of compensation or remuneration of CPs. UHC was the top ranked mean of financing CMTM model experts believe would be suitable for a mean of 2.24, third party payer was at the 2^nd^ rank with a mean of 2.45 and ″direct billing″ was at the3^rd^ rank with a mean of 3.34. However, the lowest rank was given to ″Incident to service″ with a mean of 6.76. The ICC test yielded excellent agreement among experts for this theme with a value of 0.98 (<0.001).

#### Stability (Wilcoxon signed rank test)

Wilcoxon signed rank test results denoted stability in the response of experts between 1^st^ and 2^nd^ round with a p > 0.05. Thus, null hypothesis was accepted which stated that there is no difference between the response in 1^st^ and 2^nd^ round.

## Discussion

This study deployed a modified Delphi method to present a consensus based recommendations to restructure the healthcare system at primary care level towards a more collaborative working model involving CP and GP for chronic disease management in Malaysia.

The study findings revealed significant recognition by the three group of stakeholders on the need for a CMTM model. Experts in Malaysia had complete realization of the current situation of medicine misadventures, lack of adherence, no prescription review system in private primary care especially for chronic diseases, and thus there existed high level of consensus on proper education and adherence support for chronic disease patients through an effective prescription review system in private setting and connecting all health professionals to one another through a national electronic record system where CP is viewed as reliable partner for medication management, documentation and follow up in chronic disease.

Experts also find priority areas of collaboration where need of collaboration is crucial and CMTM model may significantly benefit the Malaysian population i.e., hypertension, diabetes and asthma as top three chronic disease.

Generally, there was high level of consensus for most of the statements pertaining to structural, functional and regulatory aspects of CMTM. However, for the statements where consensus was not achieved among the experts (conflicted statements) there could be many reasons, for instance, the heterogeneity within the panel (we had quite a heterogenous panel) and nature of certain issues where high conflicts exist driven by either political rivalry or some financial conflict of interest. For such cases, expecting a high level of consensus (more than 80% agreement) would not be realistic. For example, study established that experts representing GPs hold high consensus level on the need of collaboration between CP and GP for managing chronic diseases therapy, but when next statements linked this collaboration with dispensing separation, they see it through a different lens. Thus, statement which linked collaboration with dispensing separation did not have high level of consensus but hold a moderate consensus. However, still more than 60% experts were of the view that collaboration without dispensing separation would not yield optimal results.

To authenticate the findings of this Delphi study, consensus was measured after confirmation of stability in the response of experts between two rounds. There were insignificant changes (Wilcoxon signed rank test results) in the responses of experts between 1^st^ and 2^nd^ round which means that any next round would not contribute significantly on the level of consensus. The time taken by the experts is a potential indicator of the diligence and interest of experts with which they filled the survey. The response rate in 2^nd^ round (100%) warranted that attrition was not a limitation of this study.

Our findings are in line with the results of a study carried out in Malaysia to understand the GP’s perspective about the possible extended roles of CP in patient care [53]. This study concluded a favourable response from GPs on the extended roles of CPs in patient care. It also highlighted GP’s perceived barriers in collaboration and their doubts on the clinical skills of CPs. Our study took a step further and utilized a unique method to involve experts’ panel to first gauge the current level of consensus among different healthcare stakeholders in Malaysia for active involvement of CPs in medication therapy management of chronic diseases and then engaged experts to offer consensus based solutions to various GP’s perceived problems or barriers mentioned in previous research [53] which hamper an effective collaboration between CP and GP. For example, the apprehension of GPs on the clinical skills of CPs was mentioned as a barrier in collaboration in the mentioned study. To address this apprehension, expert panel offered a consensus based solution of clinical skills enhancing course for all CPs as an essential requirement for accreditation of CPs (who intend to offer CMTM services).

On the same node, another survey based research attempted to collect GP’s views on various possible roles a CP might offer in medicine management if there is an extension in roles of CPs in Malaysia [54]. The objective was to evaluate which roles of CP are viewed positively by the GPs. With a 73.4% response rate, more than 50% of GPs were positive for CP’s roles, such as, advice on medicine, patient education and counselling, referring patient to CP in case of any drug misadventure. However, GPs have issues with separation of dispensing and prescription and change in the therapy of medication. Our study looked the same problem through a different angle and collected diverse perspectives on different roles of CP in CMTM (patient care). However, the fundamental difference is the choice of sample. Our sample was a balanced mix of experts from GPs, CPs, and Nurses. Nonetheless, our study can add to the findings that the acceptance of CP’s different roles, has been increased at least in experts’ circles, however, dispensing separation remains a burning issue. Our study finds, GPs are positive for collaboration with CPs, however, 37% experts (purely from GPs side) still believe collaboration without dispensing separation would be the only acceptable options for GPs.

The results of our study are broadly comparable to a recent international Delphi study published with a focus to push chronic care forward through collaboration among healthcare professionals in Abu Dhabi [55]. The study objectives aimed to inform the UAE’s 2021 agenda to design a world class healthcare system by setting priorities and identifying barrier in delivery of optimal chronic care in Abu Dhabi through experts’ consensus. Like our results, they also identified lack of adherence support, proper patient education, monitoring, documentation and follow up through a centralized record system as the top barriers in continuity of chronic care. The top priority in chronic care was the reorganization of healthcare system to a more patient centred approach where every healthcare provider has the access to patient information through a centralized system (an electronic record). Similar was the case in United Arab Emirates where a modified Delphi study evaluated medicine management practices for the elderly and concluded on the need of an effective collaboration between pharmacist and GP [56]. These recent updates implied even in the developing countries like Abu Dhabi or United Arab Emirates, there is an awakening to respond to the burden of chronic disease through the concept of collaborative care.

### Recommendations

This study offered potential strategies as way forward to address the problem of lack of collaboration between CP and GP in Malaysia. Based on study findings and experts’ consensus salient recommendations are being proposed for Ministry of health, Ministry of education and leadership of CPs and GPs in Malaysia.

### For the Ministry of Health

- Ministry should seek measures to utilize CP’s potentials in delivering patient-centred care through CMTM service.
- The government should initiate CMTM for at least one of the top three priority chronic diseases i.e., hypertension, diabetes, and asthma/COPD where experts have consensus that CMTM model would significantly contribute.
- There should be a regulatory check on the collaborative practice and it must involve Ministry of health, Malaysian Pharmaceutical Society, Malaysian Medical association and other relevant stakeholders. The regulatory body would devise continuous professional development’s points, conduct exams and issue license to practitioner CPs or consultant pharmacist to work under protocols for a specific chronic disease.
- An electronic national prescription database system should be developed under the auspices of Ministry of health to store the prescription records of all the chronic disease patients.
- This electronic database should be made assessable to CP licensed to collaborate with GP for a specific chronic disease.
- Both CP and GP should be compensated for CMTM service. The most feasible way to finance or remunerate the CP and GP through this service is UHC or third-party payers. Thus, government should make it compulsory for its citizen a have a health insurance.
- There should be a pilot study to run CMTM model to test its potential advantages, and if proven favourable, it should be gradually implemented in big cities in Malaysia to avoid any setback.
- To promote public awareness about the importance of CMTM, government should run a national level campaign, to explain the advantages of CMTM model and advertise slogans, such as ″Know your medicine by asking your pharmacist″.

### For community pharmacists’ leadership

- Community pharmacy needs to improve its infrastructure to offer such kind of collaborative services, for instance database system to store prescriptions’ record, such as computer, server, data storage software and internet.
- CPs should improve their clinical knowledge and skills for specific chronic diseases. Thus, before starting the service, CP should officially get a mandatory accredited training/diploma/course on CMTM service for a specific chronic disease (asthma, diabetes, hypertension).
- CP should improve their communication and documentation and must communicate all interventions to GP on a structured CP’s interventions form.

### For General Practitioners’ leadership

- GPs should recognise the needs of the aging population and importance of inter-professional collaboration in patient care in Malaysia.
- GPs should gradually adopt to a collaborative model of chronic care. In Malaysia, CP and GP should work together in managing chronic disease(s).
- Collaboration would not be optimal without dispensing separation thus; dispensing separation should be executed at least in major cities to break the ice for this collaboration.

### For the Ministry of Education

- Inter-professional education should be practically implemented in all medical and pharmacy colleges in Malaysia, so as the students understand each other role since start of their career.

### Blue print of proposed CMTM model

**Fig 4:**
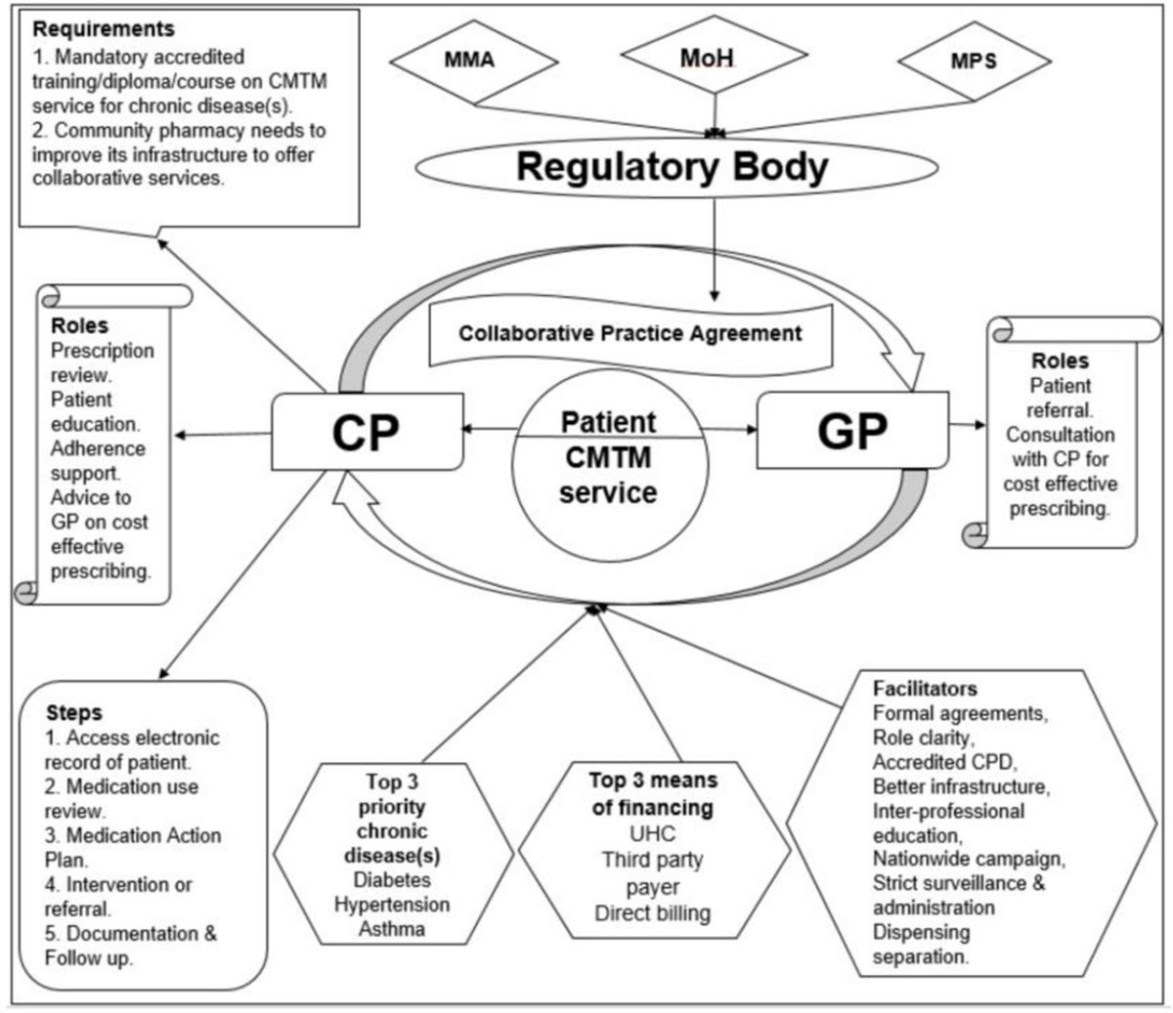
The proposed collaborative medication therapy management model. MMA = Malaysian Medical Association, MoH = Ministry of Health, MPS = Malaysian Pharmaceutical Society, CMTM = Collaborative Medication Therapy Management, CP = community pharmacy, GP = general practitioner, UHC = Universal Health Coverage, CPD = Continuous Professional Development.

The study aimed to draw a working sketch of a CMTM model, including, identifying the required resources, training, skills, accreditation, regulatory and infrastructure needs and prioritizing the common areas for collaborative practice through experts’ consensus. Based on the finding of this study we have proposed a theoretical framework for the CMTM model and is given in Fig 4.

## Conclusion

Overall, the study findings witnessed the expert panel’s support for the CMTM model for chronic care. It also explored and prioritized the issues in collaborative practice between CP and GP. The findings of the study helped to propose a working sketch of CMTM model and facilitated development of some recommendations to the authorities in Malaysia which may help to formulate a policy to bring CPs under a working relationship with GPs. Hence, this study should be taken as a call for redefining of the roles of CPs and GPs involved in primary care for chronic disease management. Undoubtedly CPs are an untapped national resource, which must be given the due role and there is acceptance of this perspective at least in the expert panel as it is the right time to share the care in Malaysia.

## Supporting information

S1 Appendix This appendix includes section A to E containing database search flowchart, search strategy, examples of articles which laid foundation of survey, survey instrument validation and complete Delphi survey.
(DOCX)

S2 Appendix This appendix includes section A to F containing tables regarding criteria of experts, qualitative comments, limitations, affiliations of experts and conflicted statements; and figures representing rating and ranking statements.
(DOCX)

S1 File This is a compressed file containing SPSS output files of median, IQR, Wilcoxon signed rank test and Kendall’s W test for both rounds. (ZIP)

S2 File This is a compressed file containing SPSS output files of ICC test of both rounds (theme-wise). (ZIP)
