# Supplementary material for: Consensus among healthcare stakeholders on a collaborative medication therapy management model for chronic diseases in Malaysia; A Delphi study
Source: PLoS One. 2019 May 10;14(5):e0216563. doi: 10.1371/journal.pone.0216563 (PMC6510413; doi:10.1371/journal.pone.0216563)

APPENDIX-I

# SECTION-A

## Database Search Flowchart


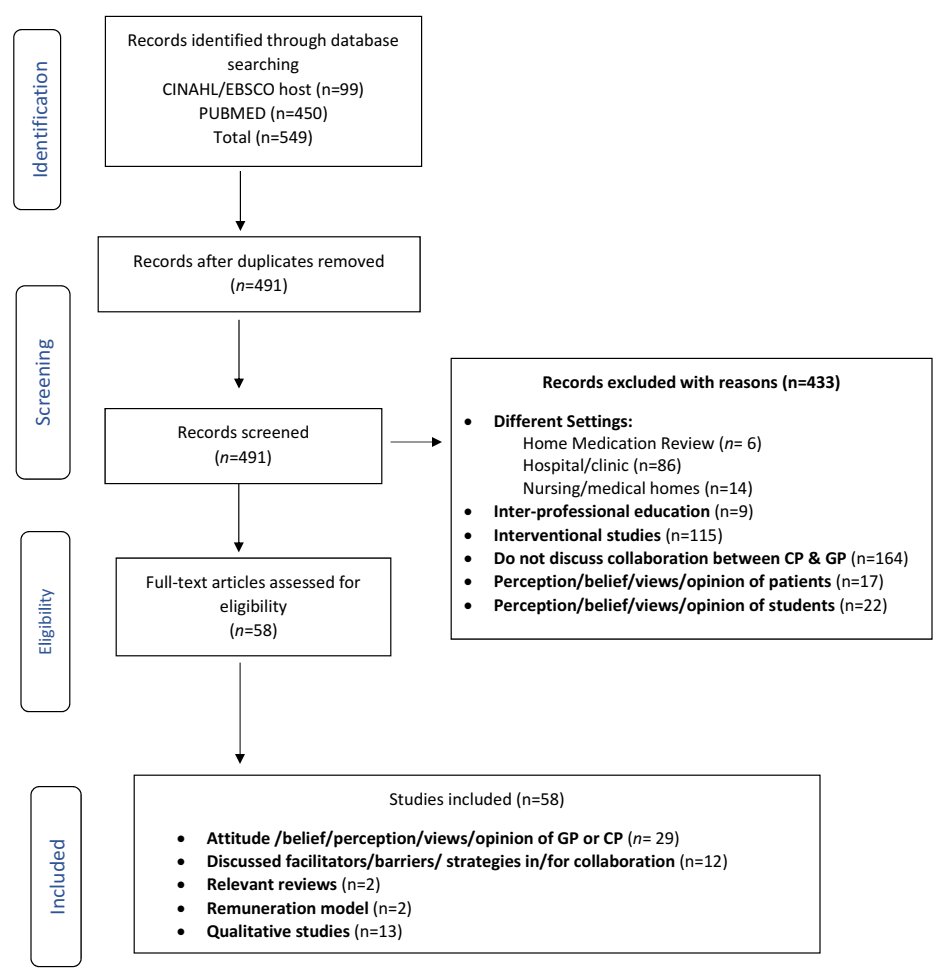


# SECTION-B

## Search Strategy

### EBSCO Host


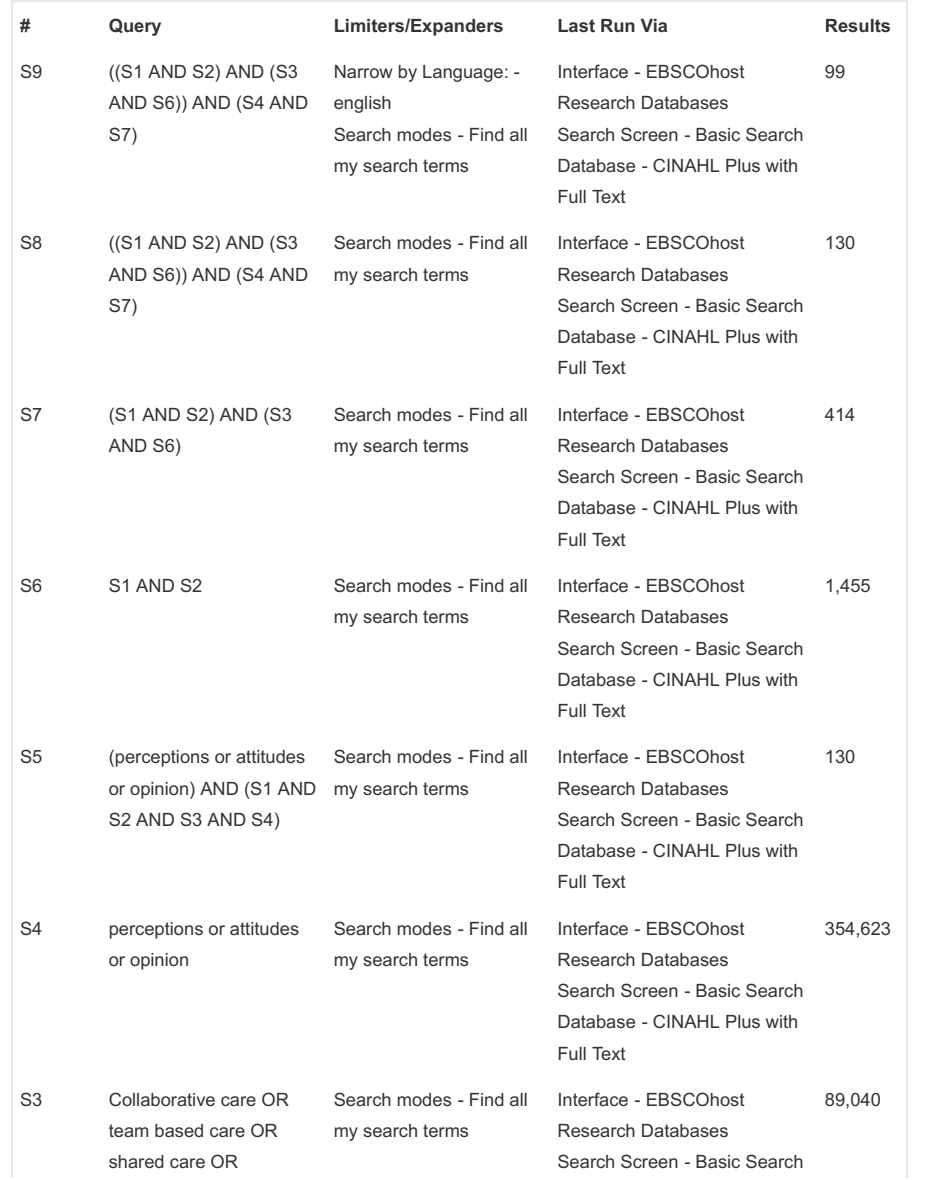


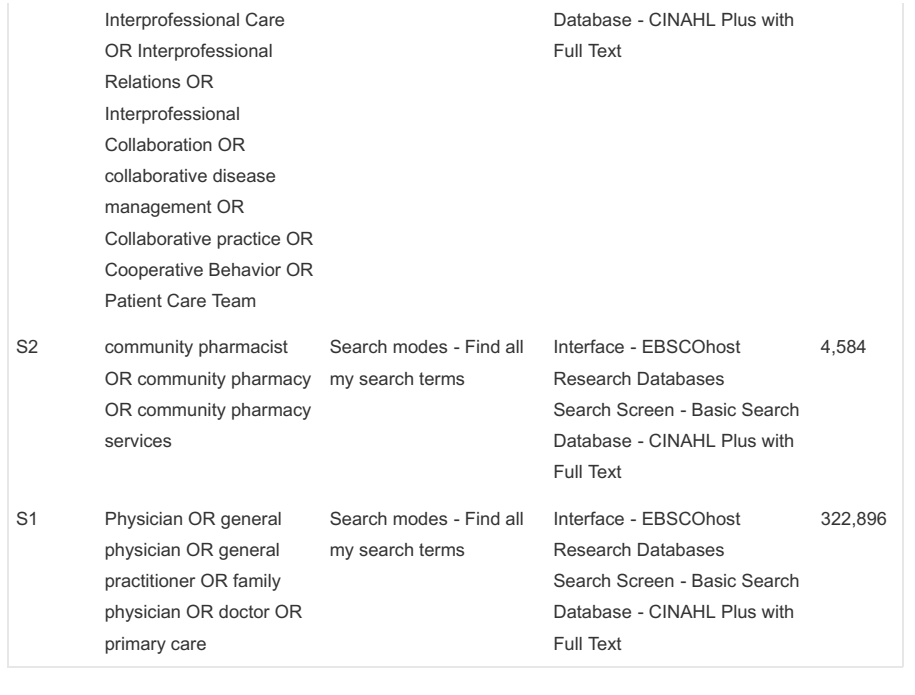


### PubMed


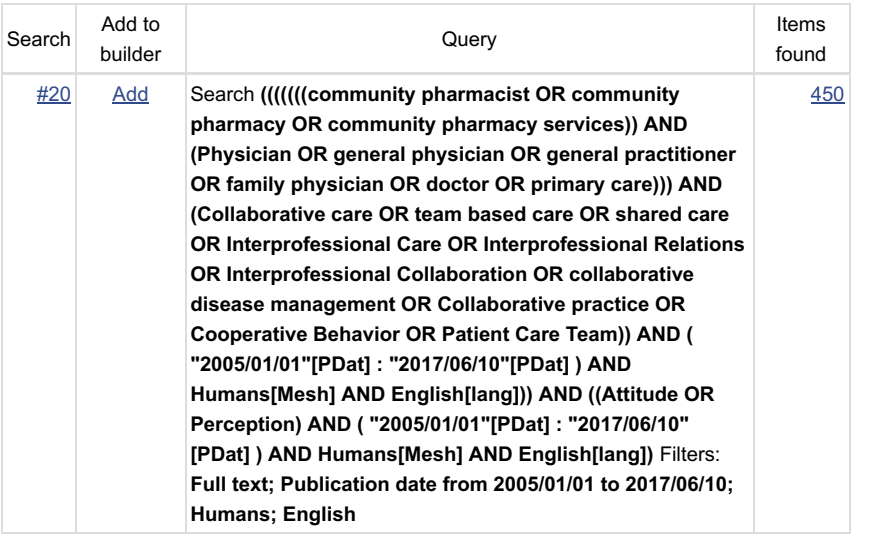


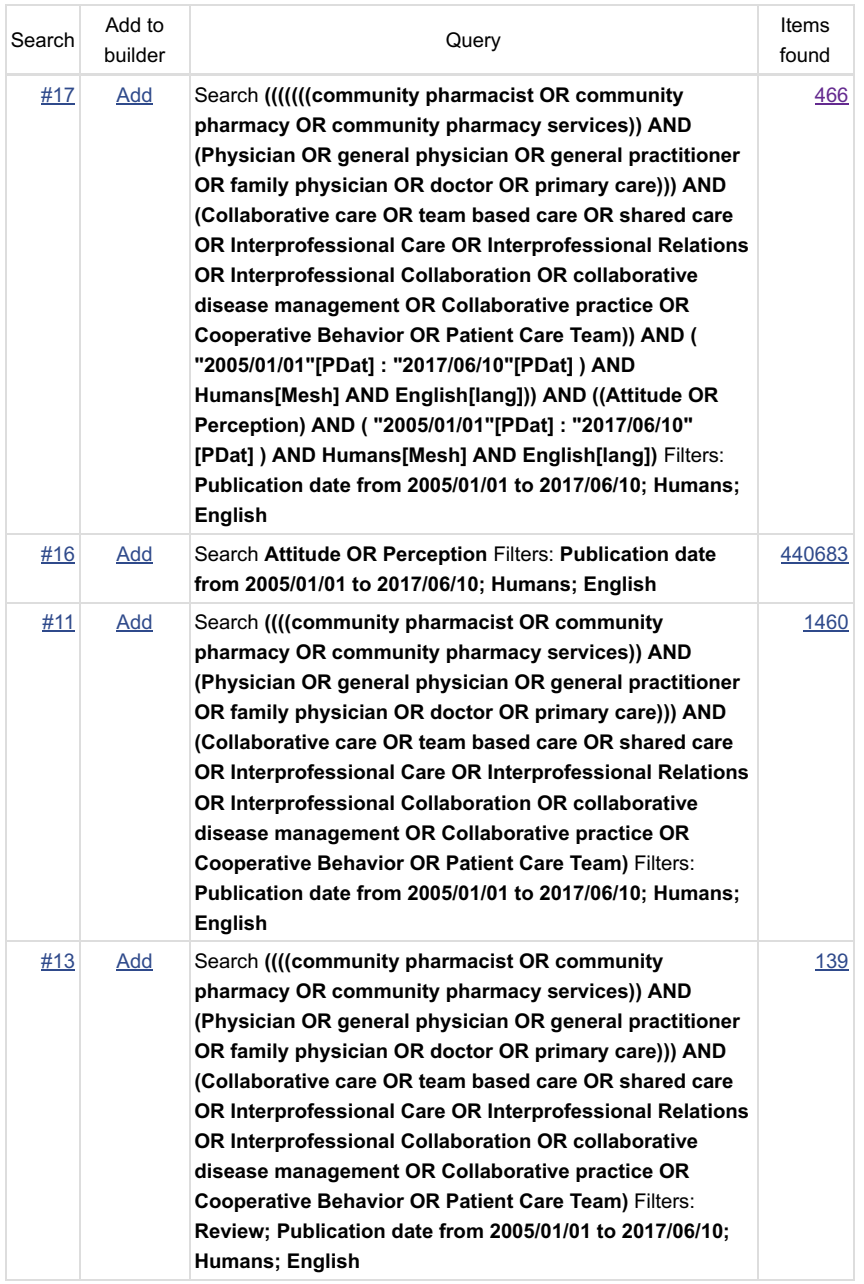


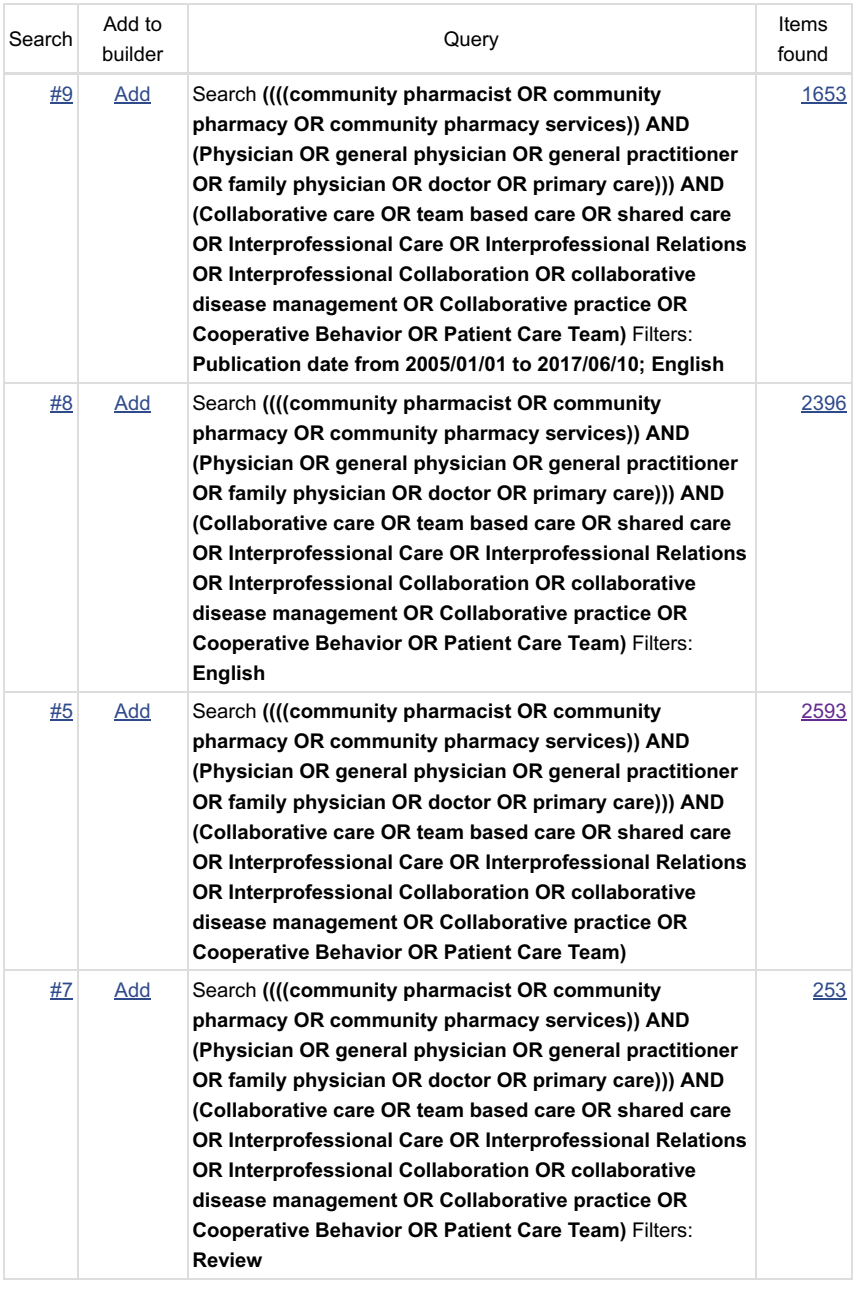


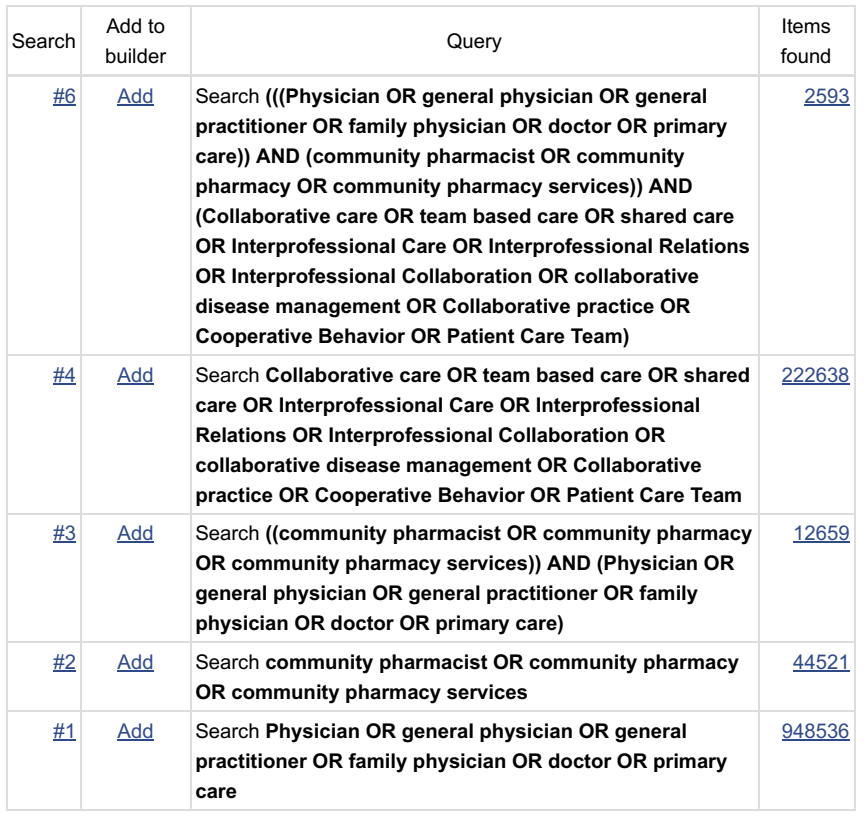


# SECTION-C

## Examples of articles that laid foundation of the survey

(Alkhateeb, Unni, Latif, Shawaqfeh, & Al-Rousan, 2009)

(Sarriff, Nordin, & Ahmad Hassali, 2012)

(Bardet, Vo, Bedouch, & Allenet, 2015; Bollen, Harrison, Aslani, & van Haastregt, 2018; Smith & Ferreri, 2016)

(Supper et al., 2015)

(D’Amour, Ferrada-Videla, San Martin Rodriguez, & Beaulieu, 2005)

(Freeman, Cottrell, Kyle, Williams, & Nissen, 2012; Loffler et al., 2017)

(Halvorsen, Stensland, & Granas, 2011)

(A, Hassali M & A, Shafie A, Saeed, 2009)

(Hassali, 2015)

(Kelly et al., 2013)

(Paulino et al., 2010)

(Avalere Health, 2015)

(Saw, Nissen, Freeman, Wong, & Mak, 2017)

(Tan, Stewart, Elliott, & George, 2014)

(Van, Costa, Abbott, Mitchell, & Krass, 2012)

(Bryant, Coster, Gamble, & McCormick, 2009)

A, Hassali M, A. A., & A, Shafie A, Saeed, M. (2009). Professional Training and Roles of Community Pharmacists in Malaysia : Views From General Medical Practitioners, *4*(2), 71–76.

Alkhateeb, F. M., Unni, E., Latif, D., Shawaqfeh, M. S., & Al-Rousan, R. M. (2009). Physician attitudes toward collaborative agreements with pharmacists and their expectations of community pharmacists’ responsibilities in West Virginia. *Journal of the American Pharmacists Association*, *49*(6), 797–800. https://doi.org/10.1331/JAPhA.2009.08111

Avalere Health. (2015). *Developing Trends in Delivery and Reimbursement of Pharmacist Services*.

Bardet, J.-D., Vo, T.-H., Bedouch, P., & Allenet, B. (2015). Physicians and community pharmacists collaboration in primary care: A review of specific models. *Research in Social & Administrative Pharmacy*, *11*(5), 602–622. https://doi.org/10.1016/j.sapharm.2014.12.003

Bollen, A., Harrison, R., Aslani, P., & van Haastregt, J. C. M. (2018). Factors influencing interprofessional collaboration between community pharmacists and general practitioners-A systematic review. *Health & Social Care in the Community*, (November), 1–24. https://doi.org/10.1111/hsc.12705

Bryant, L. J., Coster, G., Gamble, G. D., & McCormick, R. N. (2009). General practitioners’ and pharmacists’ perceptions of the role of community pharmacists in delivering clinical services. *Res Social Adm Pharm*, *5*(4), 347–362. https://doi.org/10.1016/j.sapharm.2009.01.002

D’Amour, D., Ferrada-Videla, M., San Martin Rodriguez, L., & Beaulieu, M.-D. (2005). The conceptual basis for interprofessional collaboration: Core concepts and theoretical frameworks. *Journal of Interprofessional Care*, *19*(sup1), 116–131. https://doi.org/10.1080/13561820500082529

Freeman, C., Cottrell, W., Kyle, G., Williams, I., & Nissen, L. (2012). Integrating a pharmacist into the general practice environment: opinions of pharmacist’s, general practitioner’s, health care consumer’s, and practice manager’s. *BMC Health Services Research*, *12*(1), 229. https://doi.org/10.1186/1472-6963-12-229

Halvorsen, K. H., Stensland, P., & Granas, A. G. (2011). A qualitative study of physicians’ and nurses’ experiences of multidisciplinary collaboration with pharmacists participating at case conferences. *International Journal of Pharmacy Practice*, *19*(5), 350–357. https://doi.org/10.1111/j.2042-7174.2011.00129.x

Hassali, M. A. (2015). Public Perceptions towards Implementation of Dispensing Separation: Results from a Cross Sectional Analysis. *Journal of Pharmaceutical Care & Health Systems*, *02*(04), 2–6. https://doi.org/10.4172/2376-0419.1000143

Kelly, D. V., Bishop, L., Young, S., Hawboldt, J., Phillips, L., & Keough, T. M. (2013). Pharmacist and physician views on collaborative practice. *Canadian Pharmacists Journal / Revue Des Pharmaciens Du Canada*, *146*(4), 218–226. https://doi.org/10.1177/1715163513492642

Loffler, C., Koudmani, C., Bohmer, F., Paschka, S. D., Hock, J., Drewelow, E., … Altiner, A. (2017). Perceptions of interprofessional collaboration of general practitioners and community pharmacists - a qualitative study. *BMC Health Services Research*, *17*, 1–7. https://doi.org/10.1186/s12913-017-2157-8

Paulino, E., Guerreiro, M. P., Cantrill, J. A., Martins, A. P., Costa, F. A. Da, & Benrimoj, C. (2010). Erro médico: Community pharmacists and physicians inter-professional work: insights from qualitative studies with multiple stakeholders. *Rev Port Clin Geral*, *26*(6), 590–606. https://doi.org/10.32385/rpmgf.v26i6.10802

Sarriff, A., Nordin, N., & Ahmad Hassali, M. A. (2012). Extending the Roles of Community Pharmacists: Views from General Medical Practitioners. *Medical Journal of Malaysia*, *67*(6), 577–581.

Saw, P. S., Nissen, L., Freeman, C., Wong, P. S., & Mak, V. (2017). Exploring the role of pharmacists in private primary healthcare clinics in Malaysia: the views of general practitioners. *Journal of Pharmacy Practice and Research*, 27–33. https://doi.org/10.1002/jppr.1195

Smith, M. ., & Ferreri, S. P. (2016). A model to inform community pharmacy’s collaboration in outpatient care. *Research in Social and Administrative Pharmacy*, *12*(3), 529–534. https://doi.org/10.1016/j.sapharm.2015.07.005

Supper, I., Catala, O., Lustman, M., Chemla, C., Bourgueil, Y., & Letrilliart, L. (2015). Interprofessional collaboration in primary health care: a review of facilitators and barriers perceived by involved actors. *Journal of Public Health (Oxford, England)*, *37*(4), 716–727. https://doi.org/10.1093/pubmed/fdu102

Tan, E. C. K., Stewart, K., Elliott, R. A., & George, J. (2014). Integration of pharmacists into general practice clinics in Australia: The views of general practitioners and pharmacists. *International Journal of Pharmacy Practice*, *22*(1), 28–37. https://doi.org/10.1111/ijpp.12047

Van, C., Costa, D., Abbott, P., Mitchell, B., & Krass, I. (2012). Community pharmacist attitudes towards collaboration with general practitioners: development and validation of a measure and a model. *BMC Health Services Research*, *12*, 320. https://doi.org/10.1186/1472-6963-12-320

# SECTION-D

## Survey Instrument Validation

| Sr | Statements | P-1 | P-2 | P-3 | P-4 | P-5 | P-6 | I-CVI | Verdict |
| --- | --- | --- | --- | --- | --- | --- | --- | --- | --- |
|  | In developed countries, health care systems are emphasizing much on Collaboration between GP & CP. | 3 | 4 | 4 | 1 | 1 | 1 | 0.5 | R |
|  | High incidence of drug related problems in patients of chronic diseases, made developed countries to introduce new models of community pharmacy, which focus on delivering personalized and highly accessible services like MTM to patients in Collaboration with Primary care (GP). | 3 | 4 | 4 | 4 | 2 | 2 | 0.66 | R |
|  | The new models of patient-centered community pharmacy services (which involve close collaboration between CP and GP) may help in reducing the incidence of drug related problems in patients with chronic disease(s). | 3 | 4 | 3 | 3 | 3 | 3 | 1.00 | A |
|  | Unlike many developed countries, in Malaysia, there is little collaboration between GP and CP for patient centred care. | 3 | 4 | 4 | 4 | 3 | 3 | 1.00 | A |
|  | Unlike many developed countries, in Malaysia, the potential of CP in delivering patient centred care (through MTM services) is underutilized, leading to resource wasting. | 4 | 4 | 4 | 4 | 3 | 4 | 1.00 | A |
|  | Taking example from developed countries, Malaysia should also fully utilize the CP’s potential for delivering patient cantered care (through MTM service). | 3 | 4 | 4 | 3 | 4 | 4 | 1.00 | A |
|  | In Malaysia, it is the right time to focus on Collaboration between CP and GP for advance pharmacy services i.e., MTM. | 3 | 4 | 4 | 3 | 4 | 4 | 1.00 | A |
|  | Due to increased chances of drugs interactions and adverse events/ errors. MTM like services should be proposed in developed countries. | 2 | 3 | 3 | 2 | 2 | 3 | 0.5 | R |
|  | There is a need for patient education in view of high percentage of consumers (56%), who neither understand the proper use of their medications, nor aware of their common side effect as documented in National Survey on the use of medicines by Malaysian consumers, 2012. | 3 | 4 | 4 | 4 | 4 | 4 | 1.00 | A |
|  | In the current practice of primary health care model (no CP-GP collaboration), lack of patient’s motivation is one of the reasons of poor compliance to medicines in chronic disease(s). | 3 | 4 | 4 | 3 | 3 | 3 | 1.00 | A |
|  | Poor compliance to medicines in chronic disease(s) would result in suboptimal therapeutic outcomes. | 3 | 4 | 4 | 3 | 3 | 3 | 1.00 | A |
|  | CP can propose a pharmaceutical care plan for the patients, to the GP. | 3 | 1 | 3 | 1 | 3 | 2 | 0.5 | R |
|  | Prescription review process is absent in in Malaysia’s current primary health care model. | 3 | 4 | 4 | 4 | 3 | 3 | 1.00 | A |
|  | In Malaysia’s current primary care model, many drug related problems/errors are preventable, if there exists a medicine/prescription review process (like MTM). | 3 | 4 | 4 | 4 | 3 | 4 | 1.00 | A |
|  | The overprescribing in private clinics results in increased cost of therapy to patients. | 3 | 4 | 4 | 3 | 3 | 4 | 1.00 | A |
|  | The over prescribing in private clinics introduces unnecessary medications, which increases the chances of drug-drug and drug-food interactions. | 3 | 4 | 4 | 2 | 3 | 1 | 0.66 | R |
|  | Absence of CP-GP collaboration is disadvantageous for the patients, because of missing drug expertise of CP in the form of a review of prescription (i.e., patient misses a protective layer on prescribing). | 3 | 4 | 4 | 3 | 4 | 4 | 1.00 | A |
|  | Absence of CP-GP collaboration is disadvantageous for the individual patient, because of less attention/care he receives due to less time and high number of patients waiting for single care provider (GP). | 3 | 4 | 3 | 3 | 4 | 4 | 1.00 | A |
|  | Prescriptions should be approved by CP before dispensing medications to the patients. | 1 | 3 | 2 | 3 | 3 | 1 | 0.5 | R |
|  | Absence of CP-GP collaboration is disadvantageous for the health care professionals, as they are not utilizing each other's expertise to optimize therapy. | 3 | 4 | 4 | 3 | 3 | 3 | 1.00 | A |
|  | Absence of CP-GP collaboration is disadvantageous for the Government, because lack of proper patient education on self-management and how to avoid exacerbation or adverse events, results in wastage of health care resources i.e., emergency or hospital stays. | 3 | 4 | 3 | 3 | 3 | 4 | 1.00 | A |
|  | GP and CP share common objective of improved patient care. | 4 | 4 | 3 | 4 | 4 | 3 | 1.00 | A |
|  | In Malaysia, growing health and drug related problems associated with aging population & chronic diseases can be addressed more effectively by a well-structured collaboration between GP and CP in the form of MTM. | 4 | 4 | 3 | 4 | 3 | 4 | 1.00 | A |
|  | CP may change a medicine or treatment/ give an alternative medication in case of emergency without informing GP. | 2 | 3 | 2 | 2 | 3 | 3 | 0.5 | R |
|  | CP and GP in Malaysia should collaborate for chronic disease patient’s care management. | 3 | 4 | 4 | 4 | 4 | 4 | 1.00 | A |
|  | CP may assist GP in designing drug treatment plans for patients. | 2 | 4 | 4 | 1 | 3 | 1 | 0.5 | R |
|  | CP may enhance e*vidence based medicine practice by* providing information on medicine’s use, risk, and benefits to GP | 3 | 4 | 4 | 3 | 4 | 3 | 1.00 | A |
|  | CP may have role in the prescription review which involves identifying and preventing prescription errors related to drug dose, drug-drug or food interaction or any contraindication or discuss possible alterations to medication therapy. | 3 | 4 | 4 | 4 | 4 | 4 | 1.00 | A |
|  | CP may have role in advising GP on cost-effective prescribing. | 3 | 4 | 3 | 3 | 3 | 4 | 1.00 | A |
|  | CP may improve patient care by providing advice on the use of medicine better than the GP’s assistant who do not possess appropriate knowledge and training in medicines and/or pharmacology. | 3 | 4 | 4 | 3 | 4 | 4 | 1.00 | A |
|  | CP may perform dosing adjustments to a patient’s medicine using agreed protocols established with GP. | 2 | 4 | 4 | 3 | 4 | 3 | 1.00 | A |
|  | CP may have role in ensuring patients’ medicines are effective by providing plan to GP and/or patients on potential medicine optimization such as medication adherence and others. | 2 | 4 | 4 | 3 | 4 | 4 | 1.00 | A |
|  | CP may counsel patients about what to expect from their medicine including effectiveness and relevant adverse reactions | 3 | 4 | 4 | 4 | 4 | 4 | 1.00 | A |
|  | CP may be involved in improving patients’ compliance/adherence to their medicines. | 3 | 4 | 4 | 4 | 4 | 4 | 1.00 | A |
|  | CP may supervise repeat prescriptions for a patient as per agreed protocols and contacting the GP if a problem arises. | 3 | 4 | 4 | 4 | 4 | 3 | 1.00 | A |
|  | Collaborative MTM service would engage CP-GP in an effective two-ways communication about patient drug therapy. | 3 | 4 | 4 | 3 | 4 | 4 | 1.00 | A |
|  | The two-way communication between CP -GP would results in improved patient care. | 4 | 4 | 4 | 3 | 4 | 3 | 1.00 | A |
|  | MTM offers patients and GP to take benefit from the CP’s drug expertise at a highly accessible position (community pharmacy). | 3 | 4 | 4 | 3 | 3 | 4 | 1.00 | A |
|  | MTM service (medicine review and action plan development) would improve patient’s clinical outcomes by increasing the chances for patient to achieve target therapeutic goals e.g. target blood pressure | 3 | 4 | 4 | 4 | 4 | 3 | 1.00 | A |
|  | MTM (part of education) would improve patients’ knowledge on disease and correct medicines use. | 3 | 4 | 4 | 4 | 4 | 4 | 1.00 | A |
|  | MTM would encourage GP on cost effective prescribing which leads to reduce cost of therapy for patients. | 3 | 4 | 3 | 3 | 4 | 3 | 1.00 | A |
|  | MTM (personal medication record) would serve as an early warning sign by indicating medicine usage pattern (under or over-use) of the individual patient. | 3 | 4 | 3 | 3 | 4 | 3 | 1.00 | A |
|  | MTM may significantly reduce hospital/emergency admissions by improving patients’ knowledge on self-management, and how to avoid adverse event or exacerbations. | 3 | 4 | 4 | 3 | 4 | 3 | 1.00 | A |
|  | MTM would offer an automatic prescription’s ″peer review process″ which would enhance safer prescribing practice by reducing medicine related error or inappropriate prescribing leading to improved patient safety. | 3 | 4 | 4 | 3 | 4 | 4 | 1.00 | A |
|  | The MTM (documentation and follow-up) would improve coordination of care by bringing two main stake holders in collaboration, for the benefit of the patient in a systematic way. | 3 | 4 | 1 | 1 | 4 | 2 | 0.50 | R |
|  | MTM may reduce medicine waste by improving patient’s compliance (most important factor in chronic diseases) leading to cost effective use of health care resources. | 3 | 4 | 4 | 4 | 4 | 4 | 1.00 | A |
|  | In Malaysia, CP-GP collaboration in MTM will create opportunities of transition to value-based health care system. | 3 | 4 | 4 | 3 | 3 | 4 | 1.00 | A |
|  | MTM like service by CP in Malaysia could help patients to better manage their medicine. | 3 | 4 | 4 | 3 | 3 | 3 | 1.00 | A |
|  | In Malaysia, involving CP in MTM would be an appropriate way to prevent resource waste i.e., the underutilized CP. | 3 | 4 | 4 | 3 | 3 | 3 | 1.00 | A |
|  | In Malaysia, involving CP in MTM would be an important way to improve public health outcomes. | 3 | 4 | 4 | 2 | 3 | 1 | 0.66 | R |
|  | In Malaysia, involving CP in collaborative practice would help in their own professional development. | 3 | 4 | 4 | 3 | 3 | 3 | 1.00 | A |
|  | Roles would not be clear between GP & CP | 3 | 4 | 4 | 4 | 3 | 4 | 1.00 | A |
|  | CP has more product oriented role (commercial image of CP) than patient oriented. | 3 | 3 | 4 | 3 | 3 | 3 | 1.00 | A |
|  | Concern regarding liability over shared responsibility and patient’s information. | 3 | 4 | 4 | 3 | 4 | 4 | 1.00 | A |
|  | Such collaborations would lead to GP’s role encroaching. | 3 | 4 | 3 | 3 | 4 | 3 | 1.00 | A |
|  | CP does not have professional qualification and expertise to offer such patient oriented services. | 3 | 4 | 4 | 3 | 4 | 4 | 1.00 | A |
|  | Such collaborations are threat to GP’s job (CP would steal GP’ patients and ruin the clinic business). | 3 | 4 | 4 | 3 | 3 | 4 | 1.00 | A |
|  | Compromised privacy and confidentiality in community pharmacy setup. | 3 | 4 | 4 | 3 | 4 | 4 | 1.00 | A |
|  | In Malaysia, pharmacists are not adequate to cover the aging population (shortage of CP’s manpower). | 3 | 4 | 4 | 3 | 4 | 3 | 1.00 | A |
|  | CP’s interventions would be projected as superior and a challenge to GP’s clinical decisions. | 4 | 4 | 4 | 3 | 3 | 4 | 1.00 | A |
|  | GP does not pay attention to CP’s advice | 3 | 4 | 4 | 3 | 4 | 3 | 1.00 | A |
|  | Lack of trust and appreciation from GP’s side. | 3 | 4 | 4 | 3 | 4 | 4 | 1.00 | A |
|  | Lack of incentive/remuneration for the CP. | 3 | 4 | 4 | 4 | 4 | 3 | 1.00 | A |
|  | CP is reluctant to contact GP to recommend an alternative medication. | 2 | 4 | 4 | 2 | 3 | 1 | 0.5 | R |
|  | CP has high dispensary workloads | 3 | 4 | 4 | 3 | 4 | 3 | 1.00 | A |
|  | CP do not have expertise to offer such services. | 3 | 4 | 3 | 3 | 4 | 4 | 1.00 | A |
|  | CP is not keen to take such clinical responsibilities and happy in his business. | 3 | 4 | 4 | 3 | 4 | 4 | 1.00 | A |
|  | CP is not ready to take such clinical responsibilities. | 3 | 4 | 3 | 3 | 4 | 4 | 1.00 | A |
|  | GP does not have time to discuss patient-related medicine issues with CP. | 4 | 4 | 4 | 3 | 4 | 3 | 1.00 | A |
|  | *Collaboration* between CP and GP cannot be achieved without *Dispensing separation*. | 4 | 4 | 3 | 3 | 3 | 3 | 1.00 | A |
|  | *Consumers* are not aware of CP’ such clinical roles. | 4 | 4 | 3 | 3 | 3 | 4 | 1.00 | A |
|  | Increased cost to *Consumers* due to additional CP’s consultation. | 4 | 4 | 3 | 3 | 3 | 4 | 1.00 | A |
|  | *Consumers* are still GP-centered and may not want to approach CP. | 3 | 4 | 3 | 3 | 3 | 4 | 1.00 | A |
|  | *Consumers* would not trust CP’s professional training and skills to perform such services. | 3 | 4 | 3 | 3 | 4 | 4 | 1.00 | A |
|  | Official protocol and terms of collaboration between CP and GP must be drafted and agreed beforehand. | 3 | 4 | 3 | 4 | 4 | 4 | 1.00 | A |
|  | CP and GP must be accredited (by their respective regulatory bodies) to provide the collaborative service to patients. | 3 | 4 | 4 | 4 | 4 | 2 | 0.83 | A |
|  | The service would only be provided after approval of Ministry of Health and/or Pharmaceutical division. | 3 | 4 | 3 | 3 | 4 | 3 | 1.00 | A |
|  | Patients’ recruitment in the service needs to be done through a referral system (GP to CP and vice versa) | 3 | 4 | 4 | 4 | 3 | 3 | 1.00 | A |
|  | Participation in the service must be with patients’ consent (voluntary). | 3 | 4 | 4 | 4 | 4 | 4 | 1.00 | A |
|  | The communication between CP and GP should be done through appropriate manner (such as phone conversation, referral letter, or face-to-face discussion) with at all time ensure protection over patients’ private and confidential information. | 4 | 4 | 4 | 4 | 4 | 3 | 1.00 | A |
|  | Phone could be the best way to communicate for such collaborations, topped up with two monthly face to face meeting | 3 | 4 | 4 | 3 | 3 | 1 | 1.00 | A |
|  | MTM collaborative services would be allowed only for patients with chronic disease(s), such as hypertension, asthma and diabetes. | 4 | 3 | 4 | 3 | 3 | 3 | 1.00 | A |
|  | Based on your opinion, which of the following chronic diseases may be benefited by the CP-GP collaborative practice in Malaysia? (Hypertension) | 4 | 3 | 4 | 4 | 3 | 3 | 1.00 | A |
|  | Based on your opinion, which of the following chronic diseases may be benefited by the CP-GP collaborative practice in Malaysia? Asthma/ COPD | 4 | 3 | 4 | 4 | 3 | 3 | 1.00 | A |
|  | Based on your opinion, which of the following chronic diseases may be benefited by the CP-GP collaborative practice in Malaysia? Depression | 4 | 3 | 4 | 4 | 3 | 3 | 1.00 | A |
|  | Based on your opinion, which of the following chronic diseases may be benefited by the CP-GP collaborative practice in Malaysia? diabetes | 4 | 3 | 4 | 4 | 3 | 3 | 1.00 | A |
|  | Based on your opinion, which of the following chronic diseases may be benefited by the CP-GP collaborative practice in Malaysia? AIDS | 4 | 3 | 4 | 4 | 3 | 3 | 1.00 | A |
|  | Based on your opinion, which of the following chronic diseases may be benefited by the CP-GP collaborative practice in Malaysia? cancer | 4 | 3 | 4 | 4 | 3 | 3 | 1.00 | A |
|  | As a start CPs and GPs should be allow to recruit only a certain number of patients into the service in a year. | 4 | 4 | 4 | 4 | 2 | 3 | 0.83 | A |
|  | Please rank (1-6) the following diseases in order of *Priority for Malaysian health care system,*where MTM services should be started as a first step (where 1 is TOP PRIORITY and 6 is least Priority) diabetes | 4 | 3 | 4 | 4 | 3 | 3 | 1.00 | A |
|  | Please rank (1-6) the following diseases in order of *Priority for Malaysian health care system,*where MTM services should be started as a first step (where 1 is TOP PRIORITY and 6 is least Priority) hypertension | 4 | 3 | 4 | 4 | 3 | 3 | 1.00 | A |
|  | Please rank (1-6) the following diseases in order of *Priority for Malaysian health care system,*where MTM services should be started as a first step (where 1 is TOP PRIORITY and 6 is least Priority) cancer | 4 | 3 | 4 | 4 | 3 | 3 | 1.00 | A |
|  | Please rank (1-6) the following diseases in order of *Priority for Malaysian health care system,*where MTM services should be started as a first step (where 1 is TOP PRIORITY and 6 is least Priority) asthma/COPD | 4 | 3 | 4 | 4 | 3 | 2 | 0.83 | A |
|  | Please rank (1-6) the following diseases in order of *Priority for Malaysian health care system,*where MTM services should be started as a first step (where 1 is TOP PRIORITY and 6 is least Priority) AIDS | 4 | 3 | 4 | 4 | 3 | 2 | 0.83 | A |
|  | Please rank (1-6) the following diseases in order of *Priority for Malaysian health care system,*where MTM services should be started as a first step (where 1 is TOP PRIORITY and 6 is least Priority) depression | 4 | 3 | 4 | 4 | 3 | 3 | 1.00 | A |
|  | CP and GP must not hide from each other important patients’ information that is deemed important to achieve the service’s objective. | 3 | 4 | 4 | 4 | 4 | 3 | 1.00 | A |
|  | CP must document the consultation done with patients and keep the record for review | 3 | 4 | 4 | 4 | 2 | 4 | 0.83 | A |
|  | CP must communicate to GP, all the consultation done. | 4 | 4 | 4 | 3 | 3 | 4 | 0.83 | A |
|  | To make this model work, we need to make a system of mutually agreed written standard protocol/agreement with well-defined roles, jurisdictions and terms of practice for CP and GP | 4 | 4 | 4 | 4 | 3 | 4 | 1.00 | A |
|  | Existence of formal agreements and set protocols would be an effective way for the development and maintenance of successful communication and collaboration. | 3 | 4 | 4 | 3 | 3 | 3 | 1.00 | A |
|  | These protocols would prevent concerns regarding role encroachment. | 3 | 4 | 4 | 1 | 4 | 3 | 0.83 | A |
|  | These protocols would prevent concerns regarding delivery of contradictory messages to the patients. | 3 | 4 | 4 | 3 | 3 | 1 | 0.83 | A |
|  | The motto of such protocols should be projected by the slogan of ″co-operation not conflict″ | 3 | 4 | 4 | 1 | 2 | 1 | 0.5 | R |
|  | These protocols should be established both at a professional organizations’ level (Malaysian pharmaceutical society and Malaysian medical association) and at an official level (Ministry of Health). | 4 | 4 | 4 | 4 | 3 | 3 | 1.00 | A |
|  | *Collaboration* between GP & CP can be initiated by mutual role recognition, trust, respect and motivation. | 3 | 4 | 4 | 4 | 2 | 3 | 0.83 | A |
|  | Face to face GP-CP meeting at least twice a month as a part of protocol to build rapport, trust and personal bond. | 3 | 4 | 4 | 4 | 3 | 4 | 1.00 | A |
|  | There should always be direct communication (face to face, telephone, email) and never through patients; to avoid any projection of superiority in front of patients. | 4 | 4 | 4 | 4 | 3 | 3 | 1.00 | A |
|  | Joint (CP & GP) Continuing Professional Education’s events or training could be a good way to make this *Collaboration*effective. | 3 | 4 | 3 | 4 | 4 | 3 | 1.00 | A |
|  | To build trust and understanding of roles among various professionals (medical, pharmacy, nursing), a module of inter-professional education should be part of curriculum within these professional educations. | 3 | 4 | 2 | 4 | 4 | 4 | 0.83 | A |
|  | Before starting MTM services, CP would need to officially get a specific, mandatory accredited training/ diploma/course on MTM services for a specific chronic disease (asthma, diabetes, hypertension). | 3 | 4 | 3 | 4 | 3 | 4 | 1.00 | A |
|  | Continuing Professional Development/Education for CP in the form of a mandatory accredited course/diploma can help to train CP for the changing paradigm (training to meet new health priorities, such as *MTM*). | 3 | 4 | 3 | 4 | 4 | 2 | 0.83 | A |
|  | Such training/ diploma/course for CP’s accreditation would be a joint venture Malaysian medical and pharmacy societies and ministry of health Malaysia. | 3 | 4 | 3 | 4 | 3 | 3 | 1.00 | A |
|  | Such mandatory training/ diploma/course as a professional regulation means CPs delivering MTM services have been judged against standards of clinical knowledge and competence including good communication skills with patients and other professionals. | 3 | 4 | 3 | 4 | 3 | 4 | 1.00 | A |
|  | Such trained fully accredited CP may be called ″consultant pharmacist″ or ″*Community Pharmacist* Practitioner″ would only be eligible to join these collaborative services for public. | 3 | 4 | 4 | 4 | 4 | 3 | 1.00 | A |
|  | Such accreditation of CP as ″consultant pharmacist″ would be renewed after every three years. | 3 | 4 | 4 | 4 | 3 | 3 | 0.83 | A |
|  | The current undergraduate pharmacy curriculum should be further reviewed and updated to cater public health issues with strong focus on pharmacotherapy, patient assessment skills, patient and GP centered communication skills, developing patient care plans and *MTM* skills. | 4 | 4 | 3 | 3 | 4 | 3 | 1.00 | A |
|  | Pharmacy with ″consultant pharmacist″, adequate private space and infra structure for education and counselling would only be eligible to initiate *MTM services.* | 3 | 4 | 4 | 4 | 3 | 4 | 1.00 | A |
|  | Community pharmacy needs to have appropriate national standards against which MTM service provision and the clinical care provided could objectively be judged. This would help public to make its own judgement about what to expect of the “best” services when they go to any pharmacy. | 3 | 4 | 4 | 4 | 3 | 2 | 0.83 | A |
|  | Community pharmacy should be officially categorized and advertised for its scope and area of practice (type of services it offers i.e., essential, advance) | 3 | 4 | 4 | 3 | 3 | 2 | 0.83 | A |
|  | In Malaysia, there is no shortage of pharmacist (WHO recommend pharmacist to population ratio). | 3 | 4 | 3 | 4 | 3 | 3 | 0.83 | A |
|  | High dispensary workloads of CP (involved in *MTM* services) can be reduced by delegating clerical tasks (e.g., patient identification, appointment scheduling, billing) to pharmacy technicians/ clerical staff. | 3 | 4 | 3 | 3 | 3 | 4 | 1.00 | A |
|  | As MTM involves chronic patients only and there would be a set number of maximum patients, so, it would be manageable | 3 | 4 | 4 | 3 | 3 | 4 | 1.00 | A |
|  | Malaysian Pharmaceutical Society and Ministry of Health should start promotional campaigns to educate the public on the value of such policy change. This could improve perceptions of all stakeholder groups. | 3 | 4 | 4 | 3 | 4 | 3 | 1.00 | A |
|  | To promote public awareness, Government should run a national campaign like in UK ″ask your CP about your medicine″ and what they can do for the appropriate use of medicines. | 3 | 4 | 3 | 2 | 3 | 3 | 0.83 | A |
|  | The burden of additional consultation fee for MTM services can be minimized by Government subsidies or third party payer. | 3 | 4 | 3 | 4 | 3 | 3 | 1.00 | A |
|  | For this paradigm (CP-GP Collaboration) to be successful, CP should be adequately compensated for *MTM* services. | 3 | 4 | 4 | 4 | 3 | 4 | 1.00 | A |
|  | Please rank 1-6 the most practical/feasible way of CP’s remuneration possible in Malaysia where, 1 is top most practical and 6 is least. *direct billing/ Fee for service* | 3 | 4 | 4 | 4 | 3 | 3 | 1.00 | A |
|  | Please rank 1-6 the most practical/feasible way of CP’s remuneration possible in Malaysia where, 1 is top most practical and 6 is least. *third party payer* (covered in universal health coverage or part of other health insurance coverage) | 3 | 4 | 4 | 4 | 2 | 3 | 0.83 | A |
|  | Please rank 1-6 the most practical/feasible way of CP’s remuneration possible in Malaysia where, 1 is top most practical and 6 is least. Universal health coverage (UHC) | 3 | 4 | 4 | 4 | 3 | 3 | 1.00 | A |
|  | Please rank 1-6 the most practical/feasible way of CP’s remuneration possible in Malaysia where, 1 is top most practical and 6 is least. *capitation* | 3 | 4 | 4 | 4 | 3 | 3 | 1.00 | A |
|  | Please rank 1-6 the most practical/feasible way of CP’s remuneration possible in Malaysia where, 1 is top most practical and 6 is least. *incident to service* | 3 | 4 | 4 | 4 | 3 | 3 | 0.83 | A |
|  | Please rank 1-6 the most practical/feasible way of CP’s remuneration possible in Malaysia where, 1 is top most practical and 6 is least. *Bundle payment* | 3 | 4 | 4 | 4 | 3 | 3 | 1.00 | A |
|  | Please rank 1-6 the most practical/feasible way of CP’s remuneration possible in Malaysia where, 1 is top most practical and 6 is least. *pay for performance* | 3 | 4 | 4 | 4 | 2 | 3 | 0.83 | A |
|  | Please rank 1-6 the most practical/feasible way of CP’s remuneration possible in Malaysia where, 1 is top most practical and 6 is least *cost sharing* | 3 | 4 | 4 | 4 | 3 | 3 | 1.00 | A |
|  | Government should support a national pilot trial of *MTM* services based exclusively in community pharmacy. | 3 | 4 | 4 | 4 | 2 | 4 | 0.83 | A |
|  | To avoid any economic setbacks, the policy can be gradually implemented in major cities in Malaysia. | 3 | 4 | 4 | 3 | 4 | 2 | 0.83 | A |
|  | *Collaboration* between CP and GP can be achieved even without *Dispensing separation* as it does not matter where a patient is getting medicines because at the end, he would be seeing a CP. | 4 | 4 | 4 | 4 | 3 | 3 | 1.00 | A |
|  | The objective of this collaboration is not selling drugs to get profit but improving public health outcomes and reducing cost of therapy. | 4 | 4 | 3 | 4 | 3 | 3 | 0.83 | A |
|  |  |  |  |  |  |  |  |  | Total A= 126 TOTAL R = 12 |
|  |  |  |  |  |  |  |  | **Average I-CVI= 0.92**  **S-CVI/ UA= 0.83** |  |

P= participant, A= accepted, R= rejected, I-CVI= item content validity index, S-CVI/UA= scale content validity index/ universal agreement.

# SECTION-E

## Complete Round-1 Delphi Survey (Spotlight)


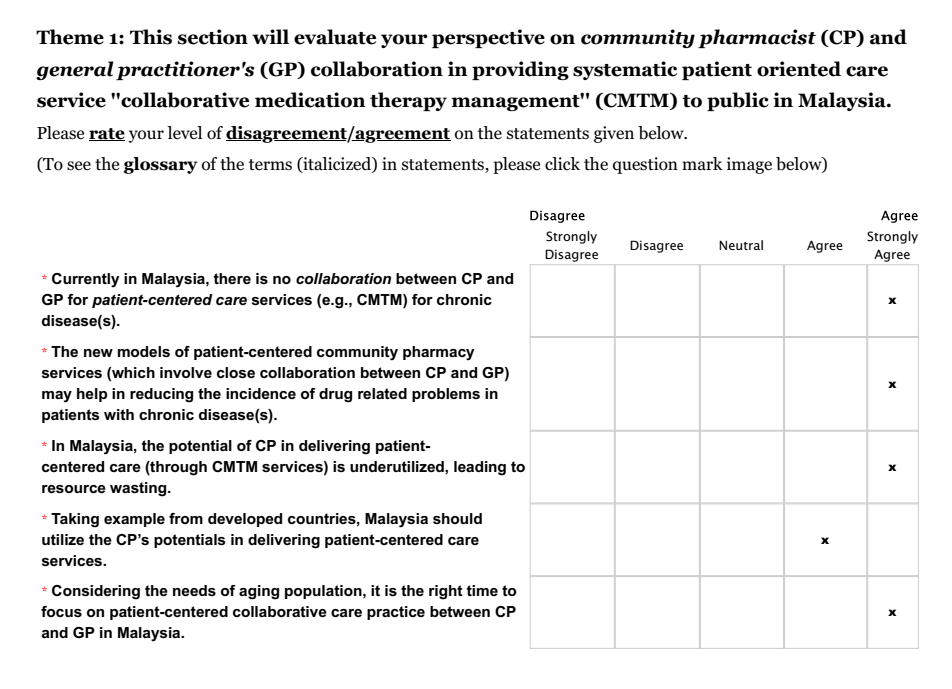


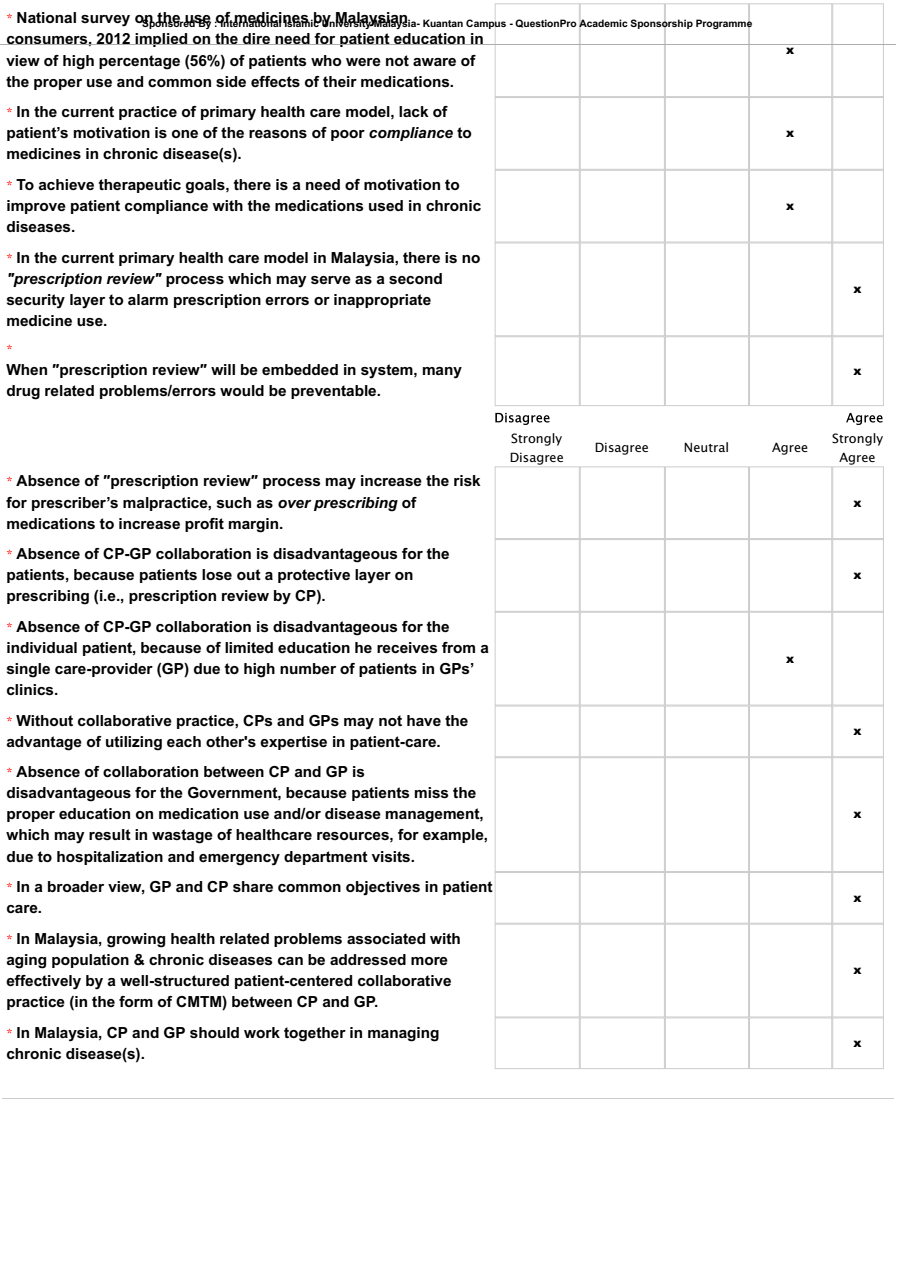


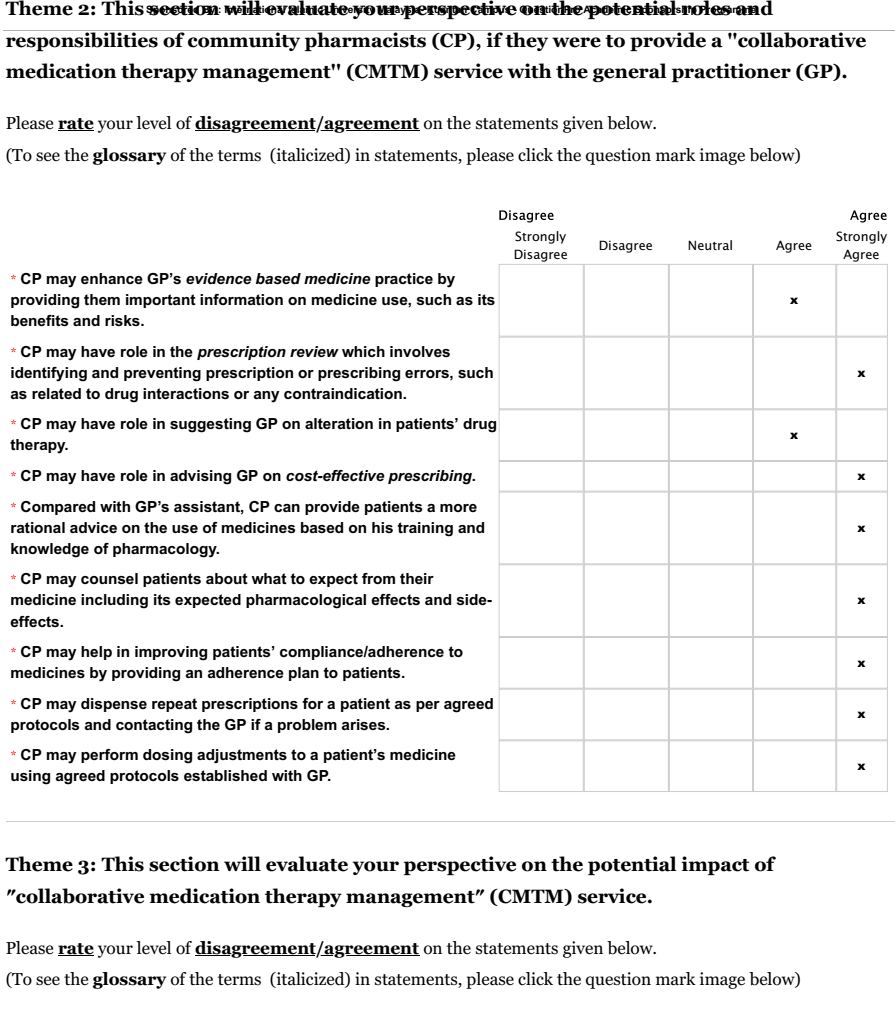


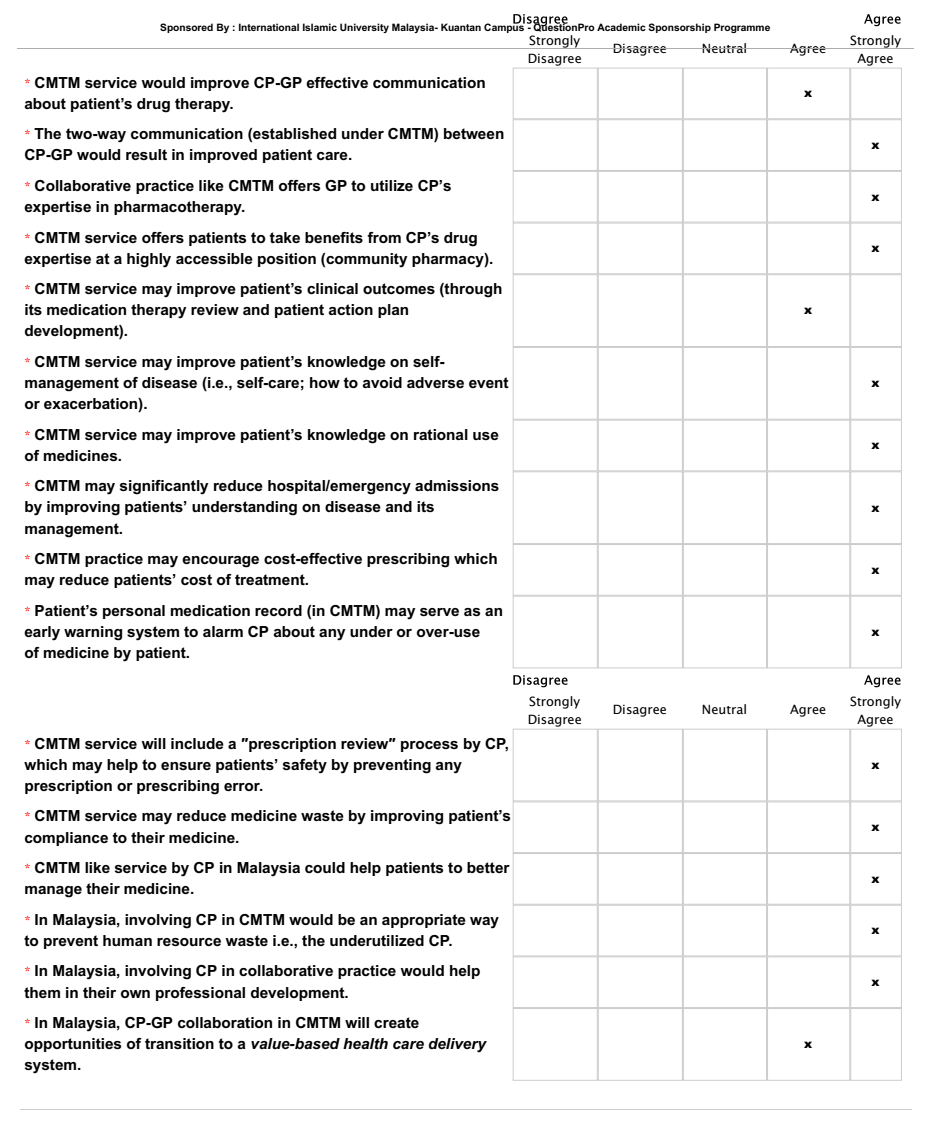


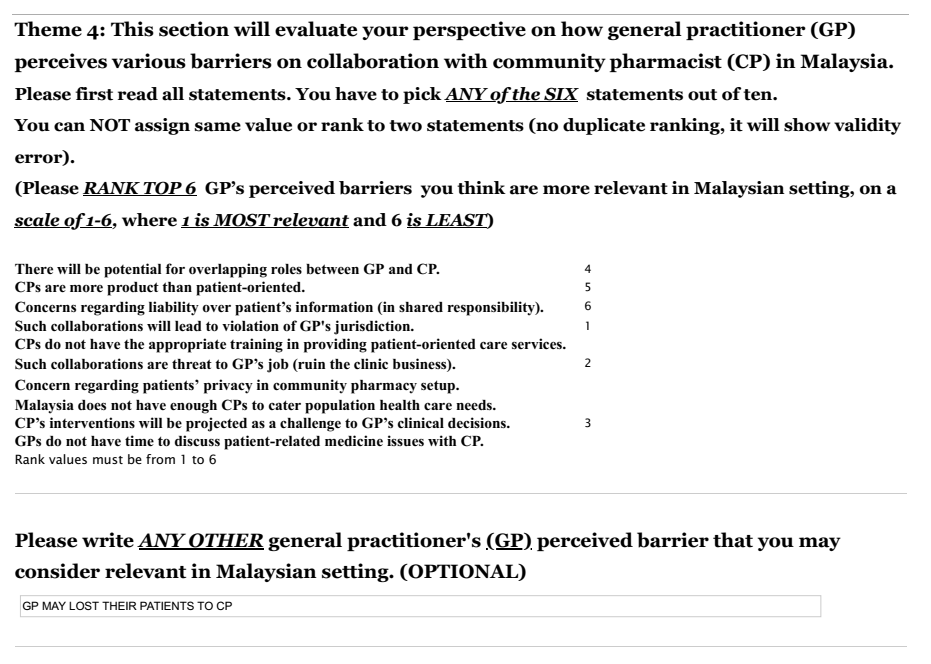


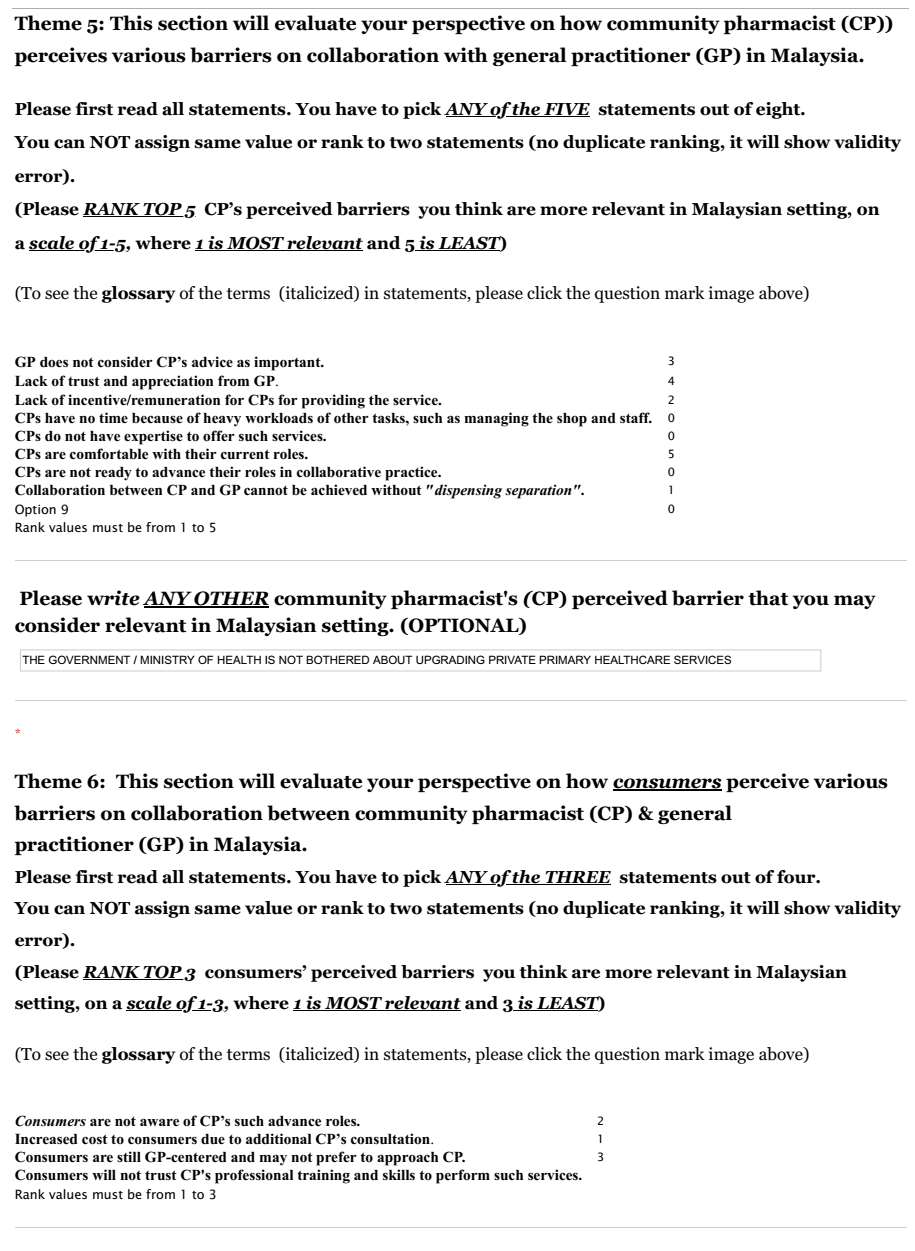


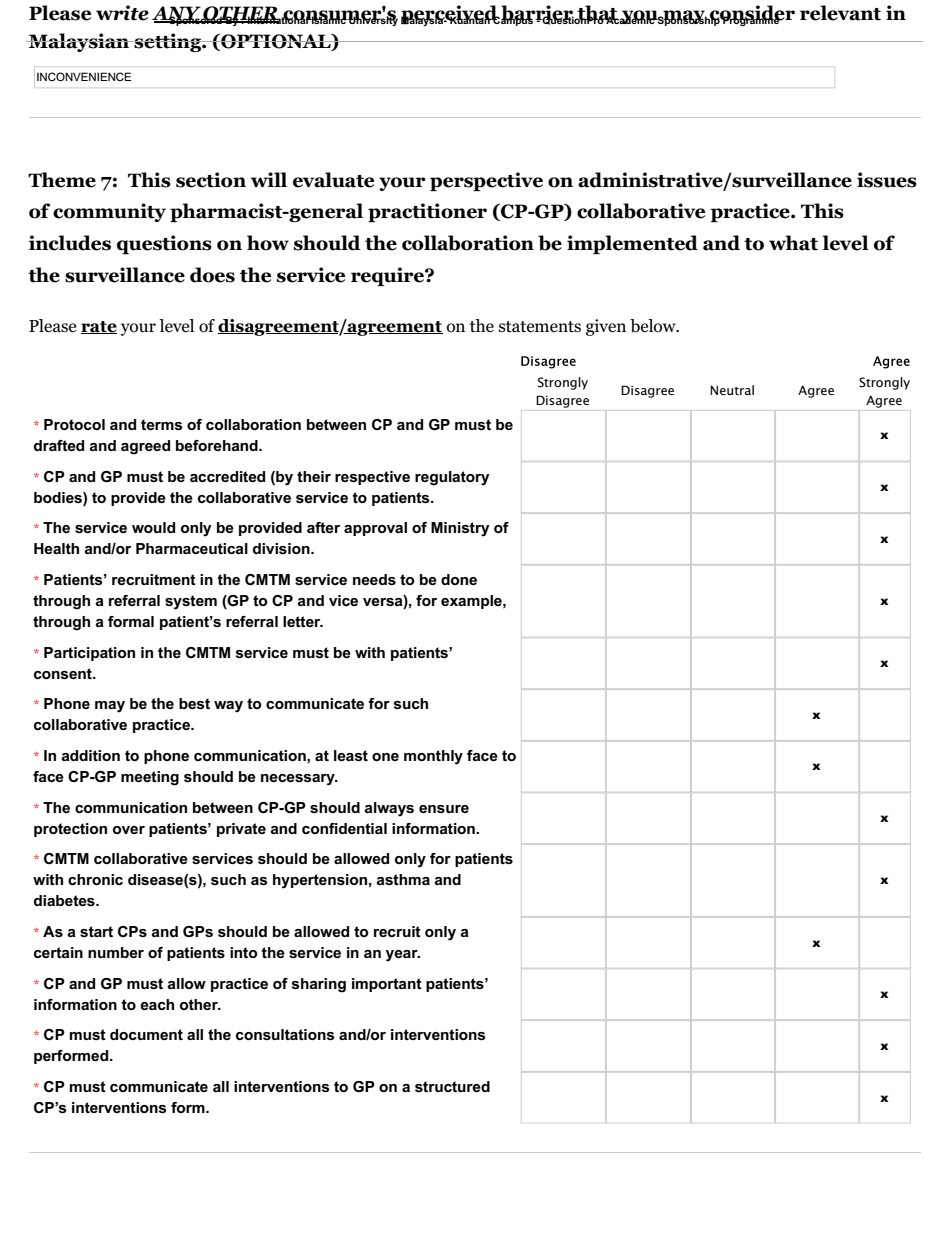


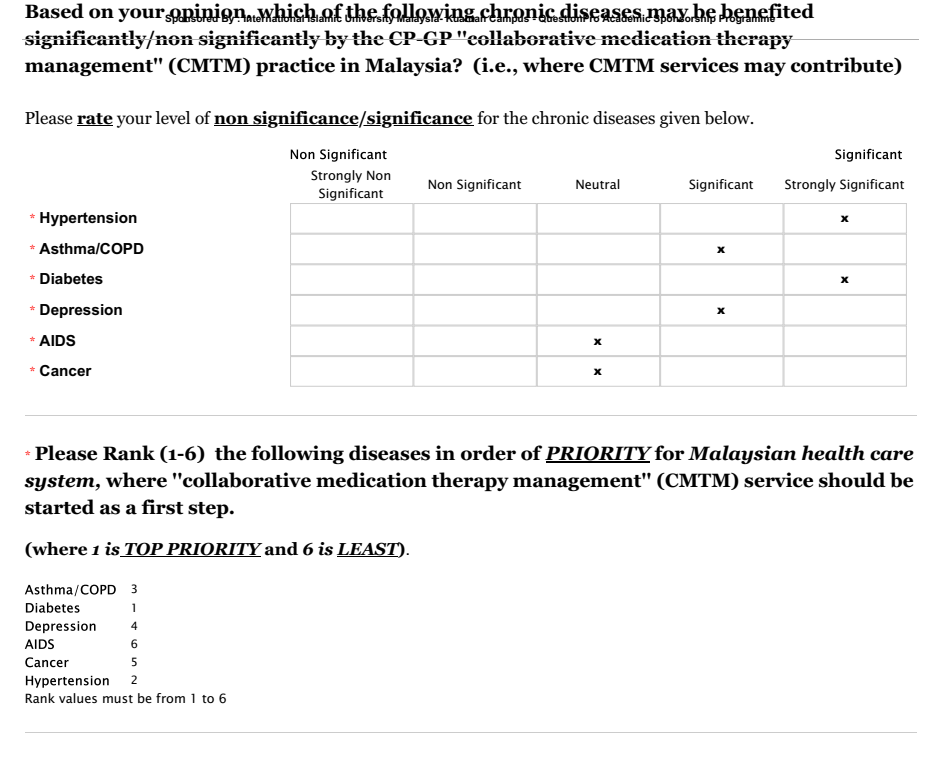


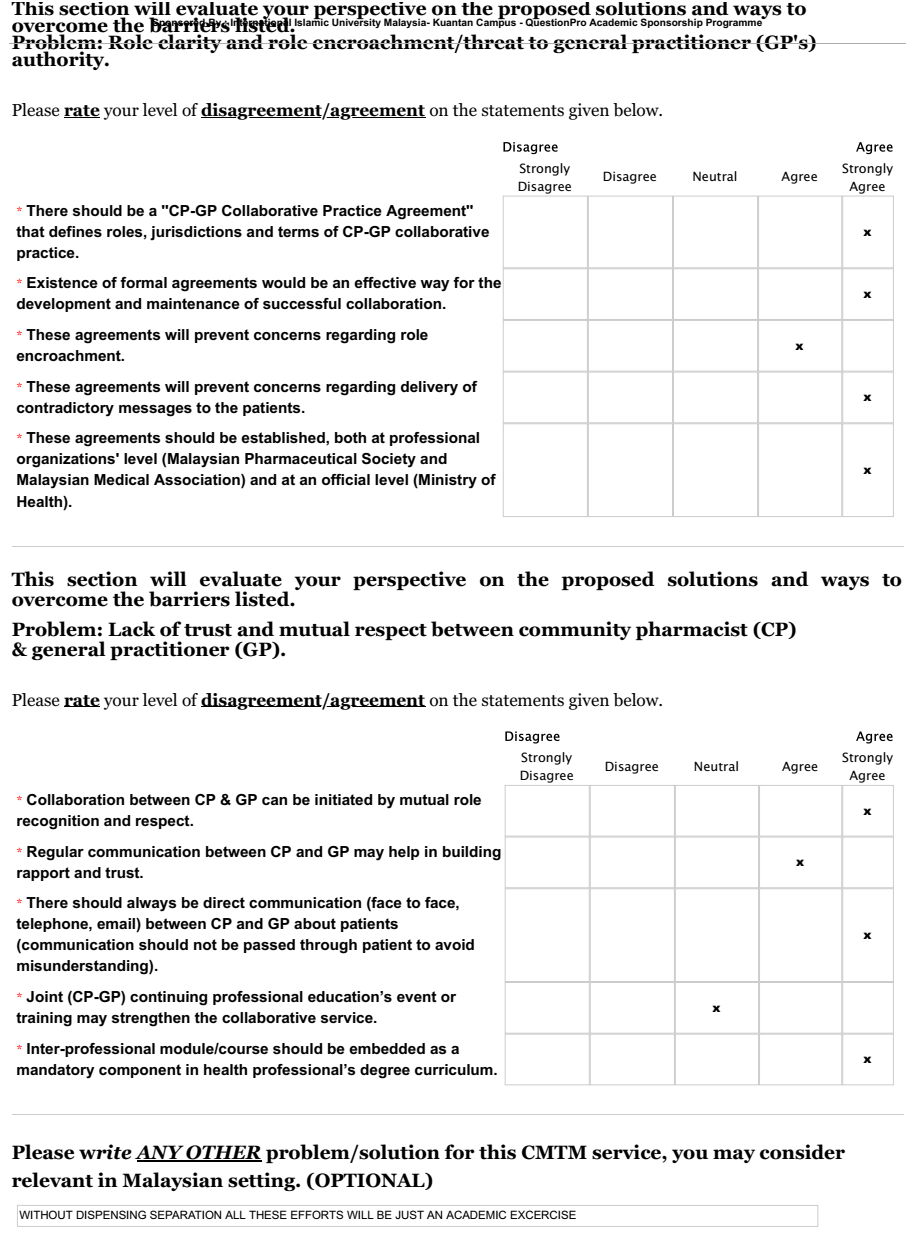


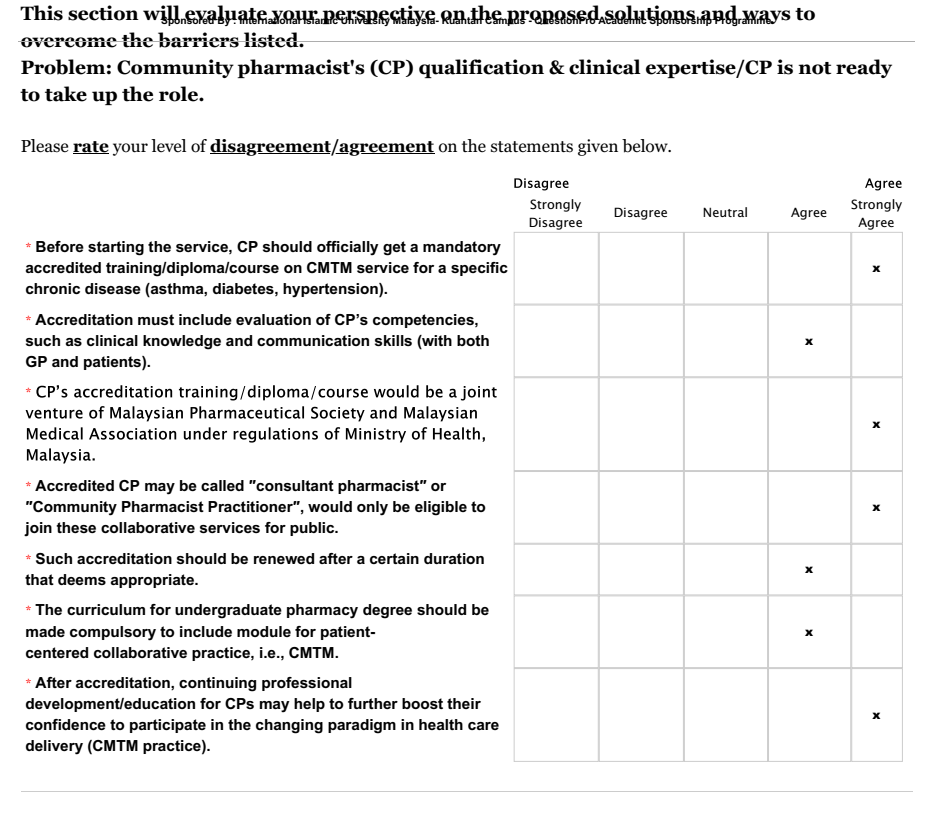


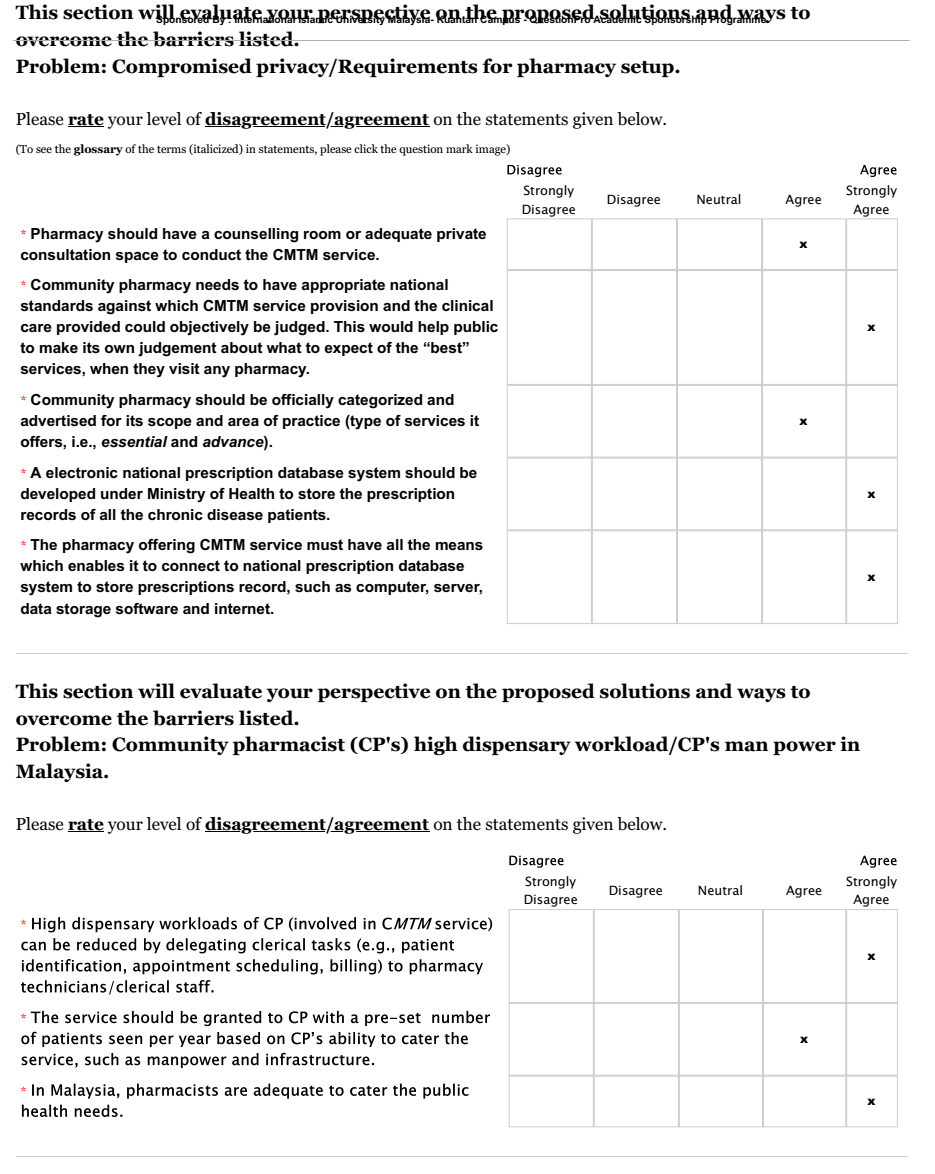


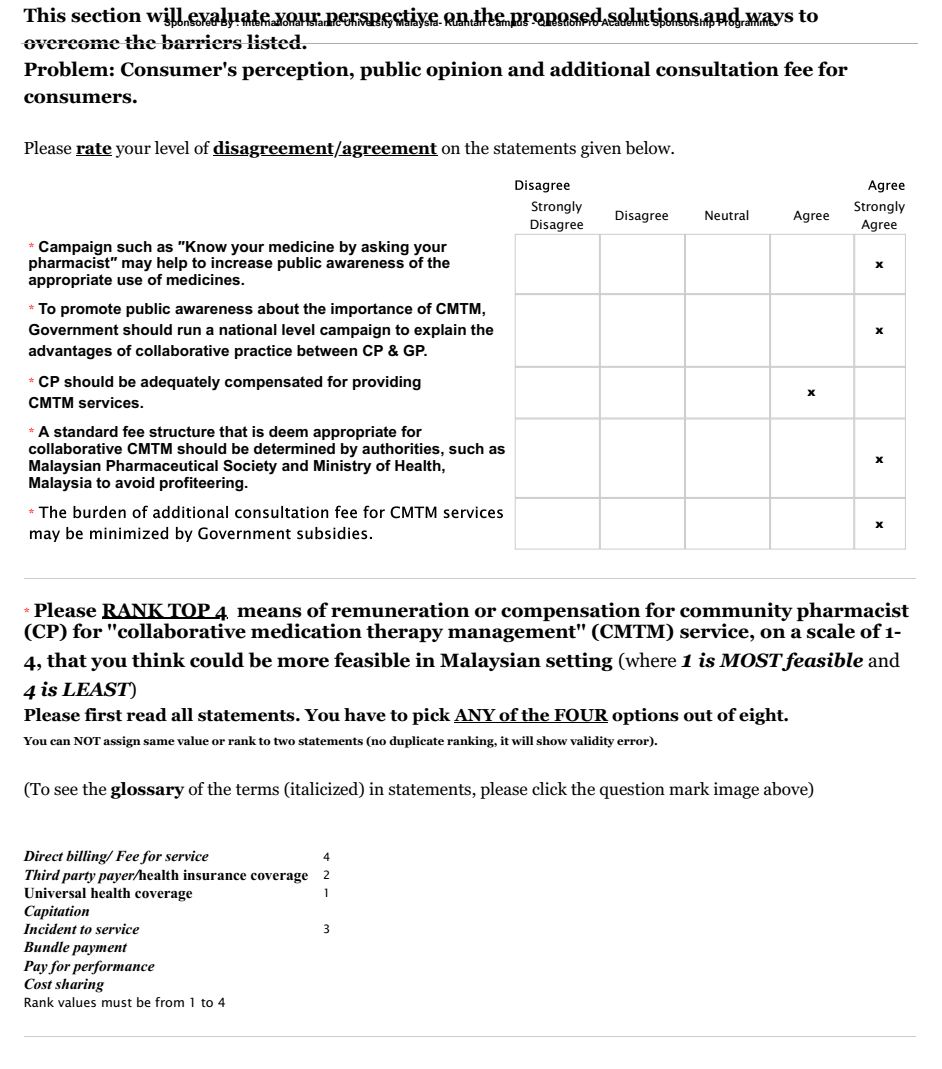


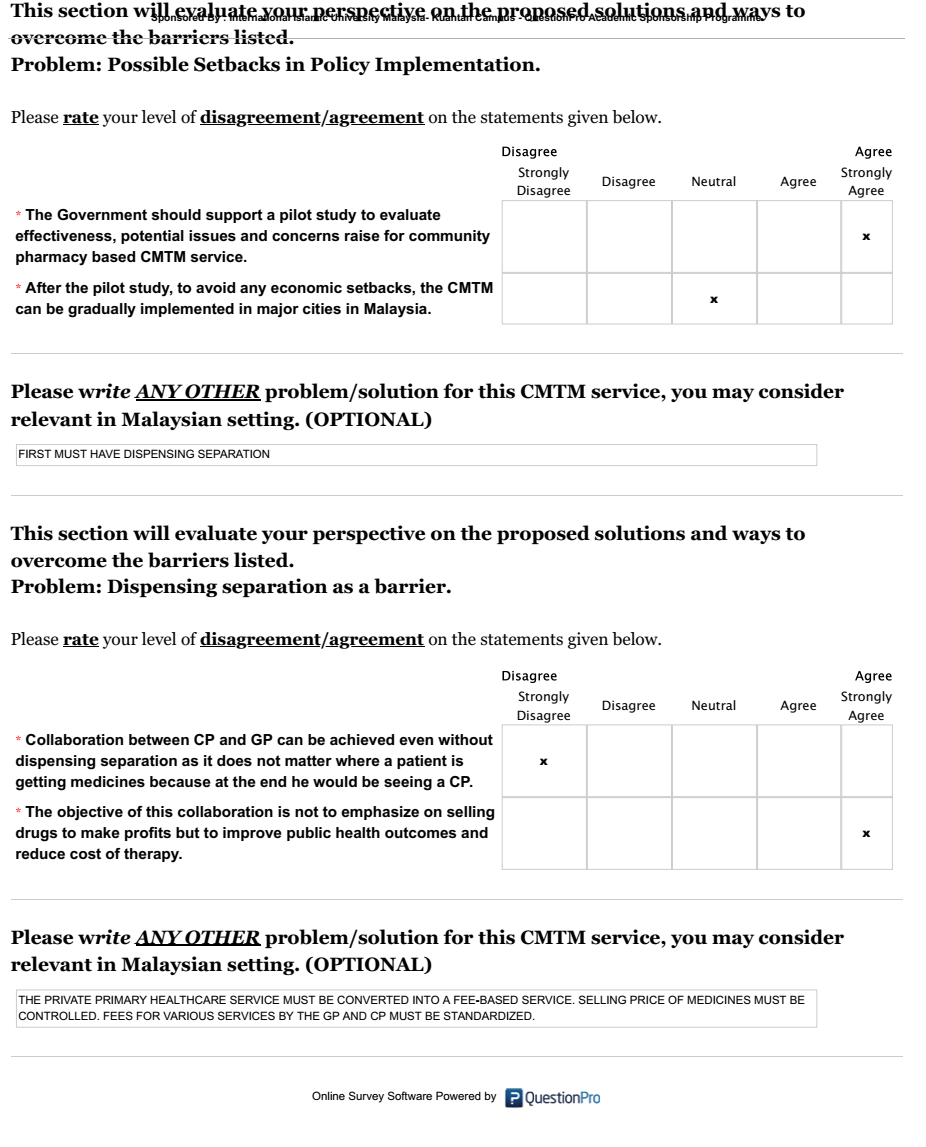


## Round-2 Delphi Survey MS-Word Document

### (Snapshot of Instructions and round-2 survey)


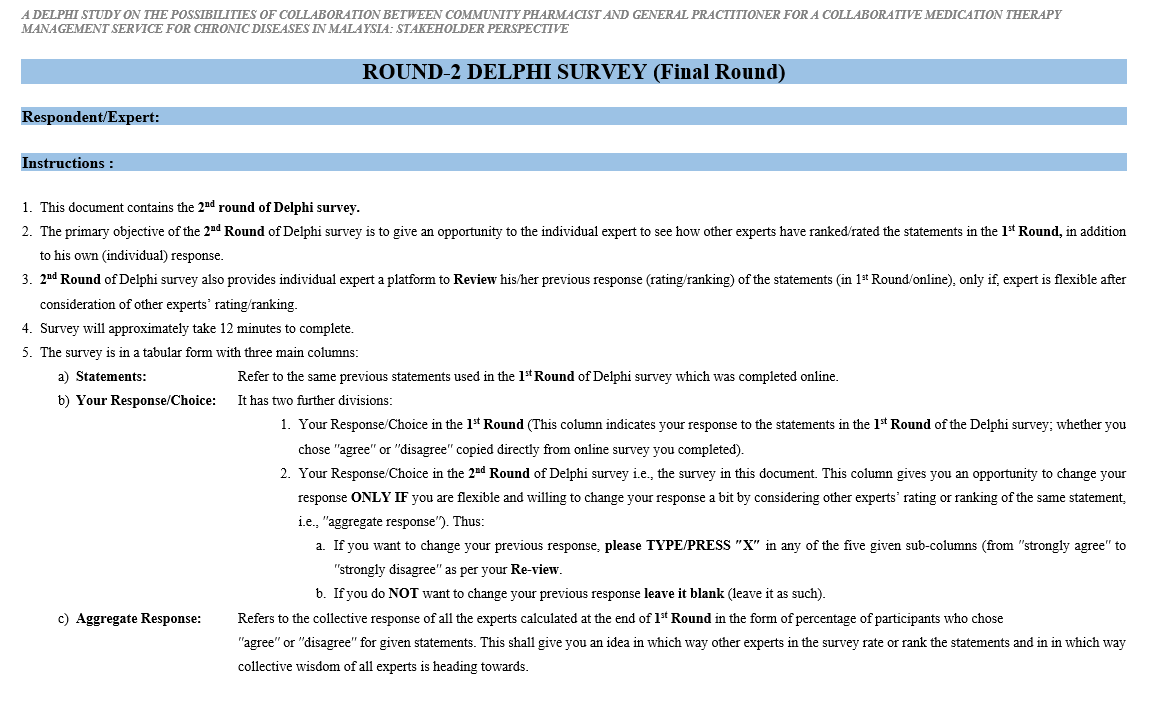


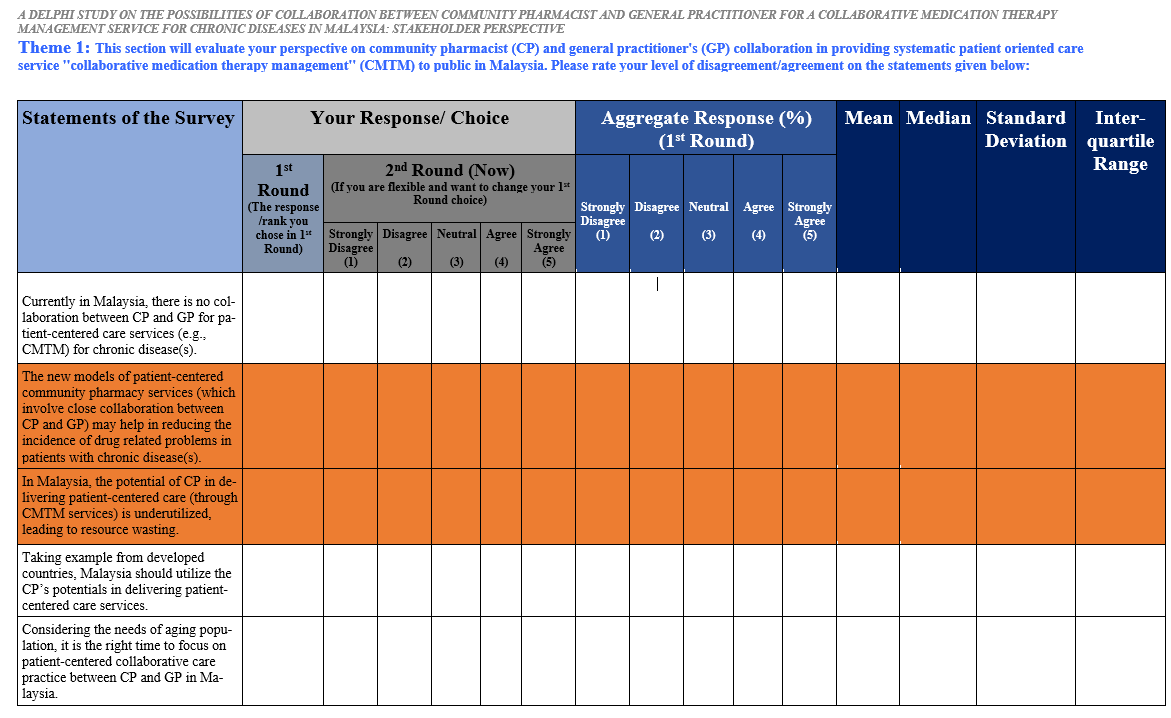

Supplement: S1 Appendix — This appendix includes section A to E containing database search flowchart, search strategy, examples of articles which laid foundation of survey, survey instrument validation and complete Delphi survey. (DOCX) [file pone.0216563.s001.docx]
