# Supplementary material for: Consensus among healthcare stakeholders on a collaborative medication therapy management model for chronic diseases in Malaysia; A Delphi study
Source: PLoS One. 2019 May 10;14(5):e0216563. doi: 10.1371/journal.pone.0216563 (PMC6510413; doi:10.1371/journal.pone.0216563)
Supplement: S2 Appendix — This appendix includes section A to F containing tables regarding criteria of experts, qualitative comments, limitations, affiliations of experts and conflicted statements; and figures representing rating and ranking statements. (DOCX) [file pone.0216563.s002.docx]

APPENDIX-II

# SECTION-A

## Criteria for Experts’ Selection

| Expert category | Criteria for experts’ selection | | |
| --- | --- | --- | --- |
|  | **Qualification/ Degree** | **Working experience** | **Experience of interaction/ working with other professionals** |
| GP | MBBS or equivalent degree. | Minimum 10 years of experience of private practice. | Minimum 3 years of experience of interactions with pharmacist in or community or hospital setting. |
| FMS | MBBS or equivalent degree with specialization. | Minimum 10 years of experience of private practice. | Minimum 3 years of experience of working with pharmacist in community or hospital setting. |
| GP in academia | Basic degree of MBBS or equivalent. Must be a PhD or equivalent degree as recognised in any of the subjects of (physiology, pharmacology, community medicine, internal medicine, oncology, endocrinology, paediatrics). | Not less than an associate professor. OR Minimum 10 years of teaching experience after basic degree. | Minimum 3 years of experience of working with pharmacist in community or hospital setting. |
| Pharmacist in MPS & pharmacy board | Basic degree in pharmacy. | Minimum 10 years of professional experience in regulatory bodies or in hospital or community. | - |
| CP | Basic degree in pharmacy. | Minimum 10 years of professional experience in community. | At least 2 years of experience of interaction with GP in hospital/clinic. |
| Pharmacist in academia | Must be a PhD (pharmacy practice, clinical pharmacy, social pharmacy, pharmacology, physiology, pharmacotherapy) with basic degree in pharmacy. | Not less than an associate professor. OR Minimum 10 years of teaching experience after basic degree. | At least have 3 years of interaction with GP in hospital/clinic. |
| Nurses in GP clinic | Basic degree in nursing. | Minimum 5 years of experience of working with GP. | Minimum 2 years of experience of working with pharmacist in community or hospital setting. |
| Nurses in nursing board | Director/Assistant director of nursing/State matron/District matron. | Minimum 10 years of experience. | Minimum 2 years of experience of working with pharmacist in community or hospital setting. |
| Nurses in academia | Master’s degree preferably PhD or equivalent with basic degree in nursing. | Not less than an assistant professor/ associate professor preferred. OR Minimum 10 years of teaching experience after basic degree. | 3 years of practice experience. |

GP= general practitioner, CP= community pharmacist, PhD= Doctor of Philosophy, FMS= Family Medicine Specialist, MBBS= Bachelor of Medicine/ Bachelor of Surgery.

# SECTION-B

## Comments (Qualitative) of Delphi experts

| **Respondent ID** | **Expert category** | **Comments** |
| --- | --- | --- |
| **Theme 4** | | |
| 26499291 | GP | ʺPharmacists in Malaysia are very commercialized, gives out prescription medicines without prescription, gives wrong advice, promote supplements over medicine/ nonevidence based therapy, doing lab tests etc. resulting GPs low trust in the pharmacists. Private patients have better chronic care control than KK patients which have pharmacists advising patients, the long queues are in KK not in private GP, patients get better advice from their GPs most of the time compared to overcrowded KKs no matter how many Dr(s) and pharmacists presentʺ. |
| 25264671 | GP | ʺThe biggest barrier to the implementation of 'Collaborative Medication Therapy Management (CMTM) would be the current healthcare financing system in in Malaysia. CMTM will never work in the private healthcare sector as there is too much competing interests between GPs and CPs. CMTM may work if Malaysia has universal health insurance coverage for the populationʺ. |
| 26064708 | GP | ʺCurrently there are cases of CF diagnosing and treating walk in patients without consulting a GP/Doctorʺ. |
| 25841371 | GP | ʺGP organizations e.g. Malaysian Primary Care Network and PERDIM will oppose to thisʺ. |
| 25864201 | GP | ʺGP is business, selling medication is part of the GP income that is why CP very difficult to establish in msiaʺ. |
| 25763981 | Nurse | ʺCost for hiring trained CPʺ. |
| 25648264 | GP | ʺLine of communication between GP and CP is not really establishedʺ. |
| 25523983 | CP | ʺLimited choice of drugs in GPs set upʺ. |
| 25438411 | CP | ʺGP may lost their patients to CPʺ. |
| 24867260 | CP | None |
| 24900646 | GP | ʺDouble charging i.e. GPs and CPsʺ. |
| 24888376 | GP | ʺDifficulty for the patient as patient needs to get the service outside GP clinic and may incur more cost and time for patientʺ. |
| 24887445 | CP | ʺLack of training as a family physicianʺ. |
| **Theme 5** | | |
| 25264671 | GP | ʺGPs perceived CPs as a threat to their professionʺ. |
| 25841371 | GP | ʺPharmacists are not interested to collaborate with GPʺ. |
| 25864201 | GP | ʺGP do not want the CP disturb their business coz this lead to low incomeʺ. |
| 25523983 | CP | ʺGP do not know the role of CPʺ. |
| 25438411 | CP | ʺThe government/ ministry of health is not bothered about upgrading private primary healthcare servicesʺ. |
| 24966946 | GP | ʺOver the counter medication hardly seen a pharmacist given proper instruction to patients.majority run by unqualified staff because the owner want to save moneyʺ. |
| 24887445 | CP | ʺLack of training in clinical skillsʺ. |
| **Theme 6** | | |
| 26499291 | GP | ʺWorried medicine not genuineʺ. |
| 25841371 | GP | ʺCustomers think about Logistic problems, where they need to go to 2 places for their consultation and drugsʺ. |
| 25683647 | Nurse | ʺCommunication pt's dataʺ. |
| 25438411 | CP | ʺInconvenienceʺ. |
| 24966946 | GP | ʺConsumer wants fast and hassle free serviceʺ. |
| **Theme 10 (Role clarity and role encroachment/threat to general practitioner (GP's) authority**). | | |
| 25264671 | GP | ʺI think the biggest threat to the private GPs will be reduction of income. GPs do not make much money from consultation under the current MMA rate. They make money from dispensing drugs. That is the main reason why do not agree to the 'separation of function' ʺ. |
| 25648264 | GP | ʺEducation and dialogue rather than an agreementʺ. |
| 25724358 | CP | ʺThe CMTM Formal Agreement should be signed only at Health Ministry Level. There is no need to duplicate it at professional organizations levelʺ. |
| 24966946 | GP | ʺCustomer maybe exposed to extra chargeʺ. |
| 24887445 | CP | ʺProvide incentives for this collaborationʺ. |
| **Theme 10 (Lack of trust and mutual respect between community pharmacist (CP) & general practitioner (GP)).** | | |
| 25264671 | GP | ʺWe must address the health financing system at the top level. CMTM may work if the financier remunerate GPs for chronic disease consultations. Without proper health financing system, patients will be reluctant to pay out-of-pocket for GPs and CPs services at the same time. It will be an extra burden to them. As it is now, many patients are already illegally buying 'prescription-only-medications (POM)' from CPs, without seeing any doctor!ʺ. |
| 25438411 | CP | ʺWithout dispensing separation all these efforts will be just an academic exerciseʺ. |
| 24966946 | GP | ʺMaybe start pilot project firstʺ. |
| 24887445 | CP | ʺEmbrace real team work in managing medications for patientsʺ. |
| **Theme 10 (Community pharmacist's (CP) qualification & clinical expertise/CP is not ready to take up the role).** | | |
| 25724358 | CP | ʺCMTM should never be accredited service. Focus should be on Pharmacy Degree Course contentsʺ. |
| 24966946 | GP | ʺShould be iso orientedʺ. |
| **Theme 10 (Qualitative Response from the Delphi Experts.  Comments of Delphi Experts on Compromised privacy/Requirements for pharmacy setup).** | | |
| 25264671 | GP | ʺWhenever possible, generic medications should be prescribed by the GPs and dispensed by the CPs under the CMTM, unless patients are willing to pay out-of-pocket for branded medicationsʺ. |
| 24966946 | GP | ʺMonitored regularly to avoid mistakeʺ. |
| **Theme 10 (Community pharmacist (CP's) high dispensary workload/CP's man power in Malaysia).** | | |
| 26499291 | GP | ʺEnsure enough pharmacists available in all areasʺ**.** |
| 25264671 | GP | ʺHow many pharmacists do we have now to cater for 32 million population? ʺ |
| **Theme 10 (Consumer's perception, public opinion and additional consultation fee for consumers).** | | |
| 26499291 | GP | ʺGPS must get appropriate consultation fees which is not happening, and this also prevents CMTM esp. due to MCOs etc. - forgot to put this as a barrier prev.ʺ. |
| 25264671 | GP | ʺCPs should also stop all the illegal dispensing of POM, without doctor’s prescriptions. This is happening rampantly in Malaysia as we speak. This is the main reason why GPs have lost trust on CPs!ʺ. |
| 25724358 | CP | ʺMalaysian Community Pharmacy Guild (MCPG) and MPS (Malaysian Pharmaceutical Society) should jointly work out a fee structure, in collaboration with Bahagian Farmasi Perkhidmatan (BPF) of MOH. The fact is these three parties (MCPG, MPS and BPF) had already worked out such a fee structure for Dispensing Charges and Drug Reimbursement Mechanism in 2015. This work was in anticipation of Dispensing Separation to be implemented in 2017 (under MOH BPF Master Plan)ʺ. |
| 24966946 | GP | ʺIt is just basically money matter, if we can be honest to public that this collaboration actually to minimize burden by both parties like overburdened, overloaded and etc. That is the bestʺ. |
| **Theme 10 (Possible Setbacks in Policy Implementation).** | | |
| 26064708 | GP | ʺPatients' rights should be addressed if the CMTM involves extra monetary expenditureʺ. |
| 25724358 | CP | ʺThere is no need to conduct such a pilot study because all the pharmacists and medical doctors in all the hospitals and clinics in Government Health Ministry are under such a CMTM for many years now. CPs and GPs in the private sector are not practicing it due mainly to fear of financial reduction and distrust with one anotherʺ. |
| 25438411 | CP | ʺFirst must have dispensing separationʺ. |
| 24966946 | GP | ʺAdvertise first to publicize their responseʺ. |
| **Theme 10 (Dispensing separation as a barrier).** | | |
| 25264671 | GP | ʺSeparation of function is the way forward. But problems with remuneration for both GPs and CPs must be addressed, as part of the universal coverage under the national health financing scheme which is long overdueʺ. |
| 26064708 | GP | ʺHow does CMTM reduces cost of therapy? ʺ |
| 25724358 | CP | ʺCollaboration between CP and GP will be almost nil if there is no dispensing separation. Both CPs and GPs will then carry on their current practice and it is the patients who will suffer the most. only government has power to implement CMTM. Health Ministry knows this fact, but the health minister lacks political will to implement dispensing separation to harmonize medication delivery differences between public and private sectorʺ. |
| 25438411 | CP | ʺThe private primary healthcare service must be converted into a fee-based service. Selling price of medicines must be controlled. Fees for various services by the GP and CP must be standardizedʺ. |
| 24966946 | GP | ʺPublic need to understand first this move otherwise the opposition will politicize itʺ. |

# SECTION-C

## Table describes these limitations and how we planned to tackle them

| Sr # | Limitation | How did we counter it? |
| --- | --- | --- |
|  | Time and resource consuming | Used e-Delphi through QP was proved to be economical  1^st^ round’s questionnaire informed by the literature. |
|  | No defined rules to conduct a Delphi study | Followed guidelines by (Boulkedid et al., 2011) |
|  | No defined guidelines to report a Delphi | Followed guidelines of (Boulkedid et al., 2011; Diamond et al., 2014) |
|  | Anonymity causes sense of isolation | Interviews of the panel in between the first and 2^nd^ round. |
|  | Non-response/ experts lose interest | Commitments of the experts were taken.  Thank you, notes,  Honorarium  Interviews of the panel in between the first and 2^nd^ round. |
|  | Non-validated questionnaire | Validation of the questionnaire is ensured before administration to expert panel |
|  | Subjectivity in defining expert, expert number (panel size), and consensus. | Followed strict guidelines  Selection of expert was under pre-defined protocol and nominations, majority of them were top leaders |
|  | Disproportionate panel of experts which can bias the result in favor of subgroup (within group) which has a greater number of representatives. | Careful selection of equal experts from all stakeholders (CP, GP and Nurse) |

CP= community pharmacist, GP= general practitioner, QP= QuestionPro.

# SECTION-D

## Field/ area of expertise and professional associations or affiliations of experts

| **Characteristics** | **Category n (%) where, n_t_=29** | | |
| --- | --- | --- | --- |
|  | **GP (n=11)** | **CP (n=10)** | **Nurse (n=8)** |
| **Your field/area of expertise *** | General practitioner 11 (42.31) | Pharmacist in academia 3 (21.43) | Nurse in academia 3 (20) |
|  | General practitioner in academia 6 (23.08) | Pharmacist in hospital 1 (7.14) | Nurse practicing with GP 7 (46.67) |
|  | Family Medicine Specialist 9 (34.62) | Pharmacist in community pharmacy 10 (71.43) | Nurse in hospital 5 (33.33) |
| **Which professional bodies have you been associated with (professional associations or affiliations)? *** | Ministry of Higher Education 3 (7.89) | Ministry of Higher Education 2 (10) | Ministry of Higher Education 5 (3.33) |
|  | Ministry of Health 3 (7.89) | Ministry of Health 1 (5) | Ministry of Health 3 (20) |
|  | Family Medicine Specialist Association Malaysia 8 (21.05) | Malaysian Pharmaceutical Society 6 (30) | Malaysian Nurses Association 6 (40) |
|  | Academy of Family Physicians Malaysia 6 (15.79) | Malaysian Community Pharmacy Guild 9 (45) | Malaysian Nursing Board 1 (6.67) |
|  | Federation of Private Medical Practitioners' Association Malaysia 3 (7.89) | Pharmacy Board/Pharmaceutical Services Division 2 (10) | - |
|  | Malaysian Medical Council 2 (5.26) | - | - |
|  | Malaysian Medical Association 2 (5.26) | - | - |
|  | Medical Practitioners Coalition Association of Malaysia 4 (10.53) | - | - |
|  | Malaysian Primary Care Network 7 (18.42) | - | - |

*Any expert could choose more than one option for field/ area of expertise and professional associations or affiliations, if might apply.

GP= general practitioner, CP= community pharmacist, n_t_= total number of experts, n= number of experts.

# SECTION-E

## Statements of 1^st^ and 2^nd^ round


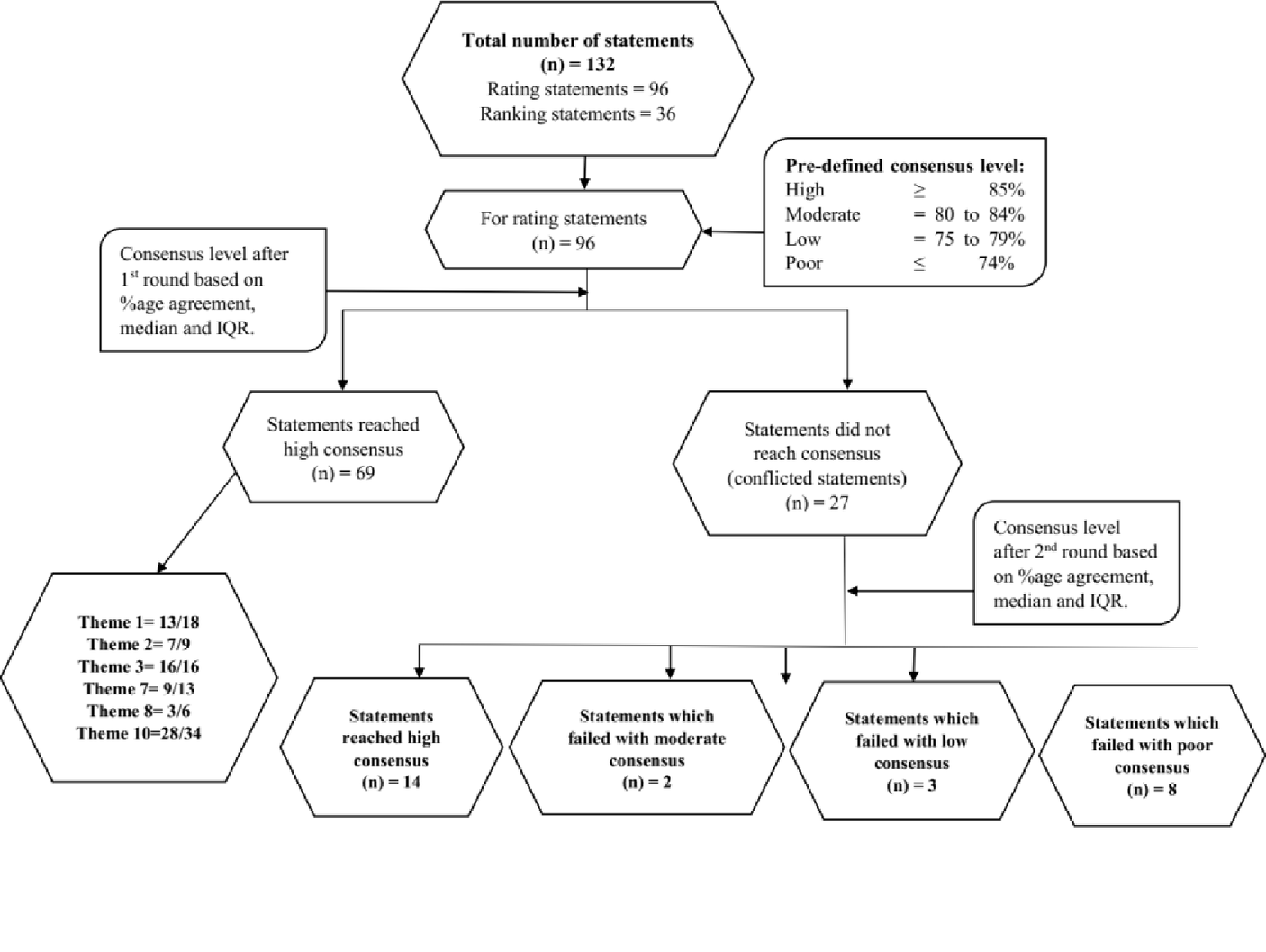


A= agree, SA= strongly agree, %= percentage, n= number of statements, IQR= interquartile range.

## Number of ranking statements


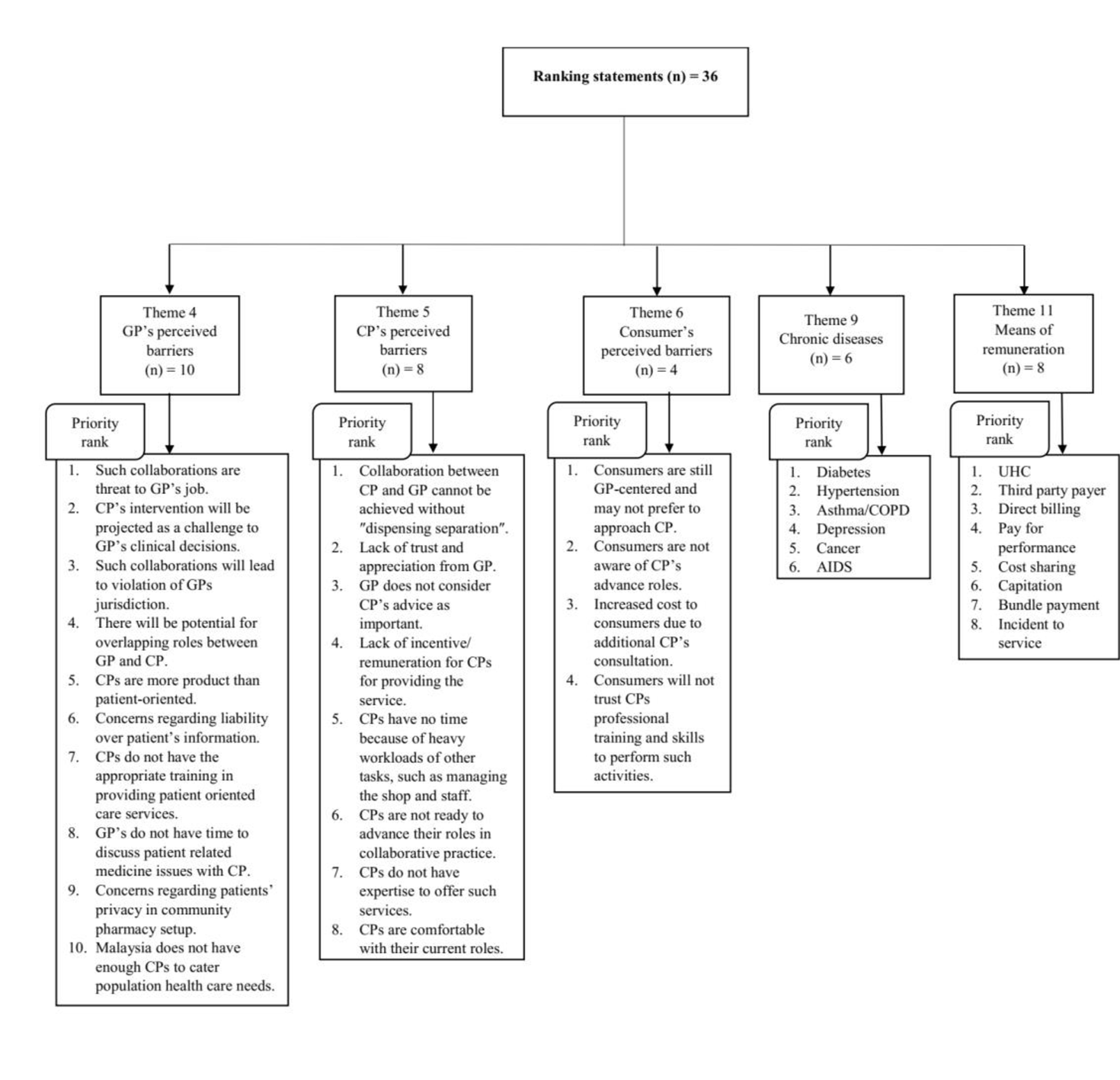


Priority rank = was established based on the final mean rank received by each statement in 2^nd^ round.

CP= community pharmacist, GP= general practitioner, COPD= Chronic Obstructive Pulmonary Disease, AIDS= Acquired Immune Deficiency Syndrome, UHC= Universal Health Coverage.

# SECTION-F

## Statements over which consensus was not achieved after 2^nd^ round

| Sr. no. | Statements | Theme | Consensus | | Comments |
| --- | --- | --- | --- | --- | --- |
|  |  |  | **1^st^ Round** | **2^nd^ Round** |  |
|  |  |  |  |  |  |
|  | In Malaysia, the potential of CP in delivering patient-centered care (through CMTM services) is underutilized, leading to resource wasting. | Theme 1 | 75.86 | 82.76 | Positive increase in consensus as it raised to approx. 7% in 2^nd^ round compared to 1^st^ round. |
|  | Absence of CP-GP collaboration is disadvantageous for the individual patient, because of limited education he receives from a single care-provider (GP) due to high number of patients in GPs’ clinics. | Theme 1 | 72.42 | 68.97 | Consensus decreased in negative direction i.e., a decrease of almost 3% was seen in consensus in 2^nd^ round. |
|  | Phone may be the best way to communicate for such collaborative practice. | Theme 7 | 62.07 | 62.07 | Consensus remained same in 2^nd^ round as compared to 1^st^ round. |
|  | In addition to phone communication, at least one monthly face to face CP-GP meeting should be necessary. | Theme 7 | 72.41 | 75.86 | Positive increase in consensus as it raised to approx. 3% in 2^nd^ round compared to 1^st^ round. |
|  | CMTM collaborative services should be allowed only for patients with chronic disease(s), such as hypertension, asthma and diabetes. | Theme 7 | 55.17 | 62.07 | Positive increase in consensus as it raised to approx. 7% in 2^nd^ round compared to 1^st^ round. |
|  | As a start CPs and GPs should be allowed to recruit only a certain number of patients into the service in a year. | Theme 7 | 75.87 | 79.31 | Positive increase in consensus as it raised to approx. 4% in 2^nd^ round compared to 1^st^ round. |
|  | Depression | Theme 8 | 51.72 | 58.62 | Positive increase in consensus as it raised to approx. 7% in 2^nd^ round compared to 1^st^ round. The level of consensus shows depression was not considered by the expert panel to be impacted by CMTM model. |
|  | AIDS | Theme 8 | 34.48 | 34.48 | Consensus remained same in 2^nd^ round as compared to 1^st^ round. The level of consensus shows AIDS was not considered by the expert panel to be impacted by CMTM model. |
|  | Cancer | Theme 8 | 41.38 | 41.38 | Consensus remained same in 2^nd^ round as compared to 1^st^ round. The level of consensus shows cancer was not considered by the expert panel to be impacted by CMTM model. |
|  | The service should be granted to CP with a pre-set number of patients seen per year based on CP’s ability to cater the service, such as manpower and infrastructure. | Theme 10 (Workload) | 82.76 | 82.76 | Consensus remained same in 2^nd^ round as compared to 1^st^ round. |
|  | In Malaysia, pharmacists are adequate to cater the public health needs. | Theme 10 (Workload) | 48.28 | 58.62 | Positive increase in consensus as it raised to approx. 4% in 2^nd^ round compared to 1^st^ round. |
|  | The burden of additional consultation fee for CMTM services may be minimized by Government subsidies. | Theme 10 (Consumer’s perception) | 75.86 | 79.31 | Positive increase in consensus as it raised to approx. 4% in 2^nd^ round compared to 1^st^ round. |
|  | Collaboration between CP and GP can be achieved even without dispensing separation as it does not matter where a patient is getting medicines because at the end, he would be seeing a CP. | Theme 10 (Dispensing separation) | 37.93 | 37.93 | Consensus remained same in 2^nd^ round as compared to 1^st^ round. |

After 2^nd^ round, n=13 statements could not establish consensus, of which (n= 7) statements achieved consensus in positive direction (increased consensus as compared to 1^st^ round), (n= 1) statement achieved consensus in negative direction (decreased consensus as compared to 1^st^ round) and (n= 5) statements consensus remained same as compared to 1^st^ round.

CP= community pharmacist, GP= general practitioner, CMTM= Collaborative Medication Therapy Management, AIDS= Acquired Immune Deficiency Syndrome.
